# Impact of training data on deep learning–based RNA 3D structure quality assessment

**DOI:** 10.1093/bib/bbag630

**Published:** 2026-11-09

**Authors:** Bartosz Adamczyk, Marta Szachniuk, Maciej Antczak

**Affiliations:** Institute of Bioorganic Chemistry, Polish Academy of Sciences, Noskowskiego 12/14, PL-61-704 Poznan, Poland; Institute of Computing Science, Poznan University of Technology, Jacka Rychlewskiego 1, PL-61-131 Poznan, Poland; Institute of Bioorganic Chemistry, Polish Academy of Sciences, Noskowskiego 12/14, PL-61-704 Poznan, Poland; Institute of Computing Science, Poznan University of Technology, Jacka Rychlewskiego 1, PL-61-131 Poznan, Poland; Institute of Bioorganic Chemistry, Polish Academy of Sciences, Noskowskiego 12/14, PL-61-704 Poznan, Poland; Institute of Computing Science, Poznan University of Technology, Jacka Rychlewskiego 1, PL-61-131 Poznan, Poland

**Keywords:** RNA 3D structure prediction, general quality assessment, deep learning, reference-free evaluation

## Abstract

Accurate reference-free assessment of predicted RNA 3D structures is essential for large-scale RNA modeling, yet the impact of training data on deep learning–based quality predictors remains poorly understood. Here, we present a systematic cross-training analysis of three deep learning predictors for RNA 3D structure quality assessment, each originally developed on distinct training sets. By retraining each predictor on alternative (nonnative) datasets—including RNA3DQA, a new set created specifically for this project—and evaluating performance on independent RNA-Puzzles benchmarks, we systematically assess the relative contributions of model architecture and training data composition. Our results reveal pronounced differences in cross-dataset generalization, with predictors often performing better when trained on nonnative datasets than on their native ones. Notably, the impact of the training datasets varied across predictors, with RNA3DQA yielding the overall best accuracy and ranking stability results specifically for the ARES model. Native training sets yielded better performance for RNA3DCNN and lociPARSE. These findings demonstrate that the characteristics of the training data play a dominant, model-dependent role in shaping the performance and robustness of reference-free RNA 3D structure quality assessment methods. We conclude with practical recommendations for designing transferable training datasets and for benchmarking deep learning–based quality predictors in RNA structural bioinformatics.

## Graphical abstract

**Graphical Abstract:**
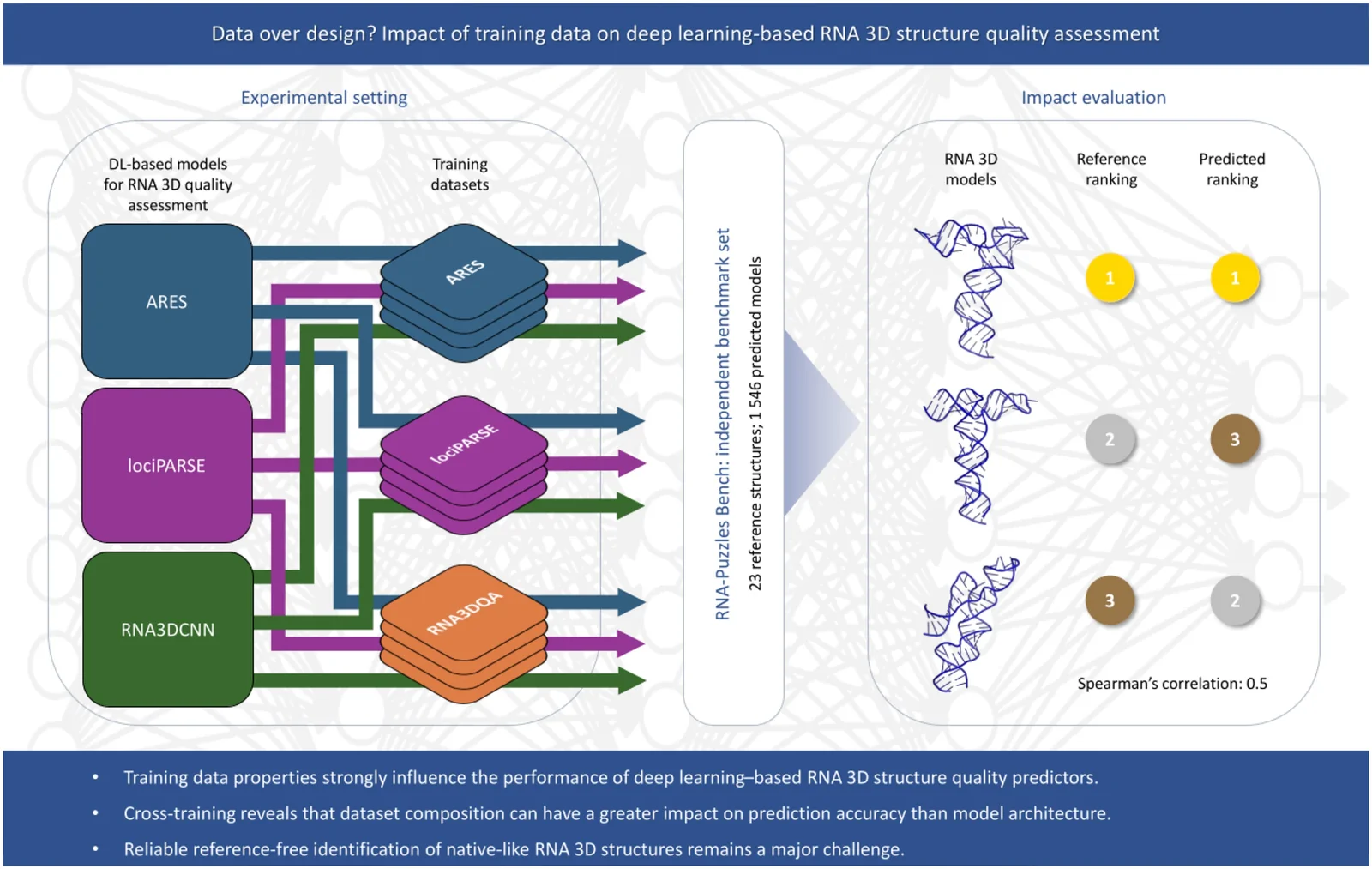

## Introduction

Computational prediction of biomolecular 3D structures has become an increasingly important component of modern structural biology, driven by the growing availability of sequence data and the limited throughput of experimental structure determination [1–4]. Many contemporary methods for RNA 3D structure prediction can generate large ensembles of candidate models, often numbering in the hundreds or thousands for a single RNA molecule [5–7]. To be practically useful, such methods must rely on reliable internal mechanisms to assess and rank predicted models, thereby enabling the selection of the most plausible structures for downstream analysis and user presentation. The importance of such quality assessment mechanisms has become even more pronounced with the rapid expansion of deep learning (DL)-based RNA structure prediction approaches, which can efficiently produce diverse structural hypotheses but may vary substantially in their predictive reliability [8–14].

Assessing the quality of predicted RNA 3D models is straightforward when an experimental reference structure is available; however, predicting a known structure does not represent a meaningful challenge in real-world applications. In practice, RNA 3D structure prediction is needed precisely when the native structure is unknown. Consequently, the ability to evaluate and rank predicted models in the absence of reference structures, using general, reference-free quality assessment methods, is essential both for selecting high-quality predictions and for estimating the reliability of computational RNA 3D structure prediction pipelines [15].

In recent years, DL-based predictors have emerged as the new approach for reference-free RNA 3D structure quality assessment. These methods exploit a wide range of structural descriptors, including geometric features, inter-residue interactions, and global conformational properties, and have consistently outperformed earlier heuristic and physics-based scoring functions. Three predictors have been proposed, differing in neural architectures, feature representations, and training strategies [16–18]. They were trained and evaluated on datasets comprising experimentally determined RNA 3D structures paired with large collections of decoy models. As a result, comparisons between methods have largely focused on architectural innovations and feature engineering, often treating the associated training datasets as fixed, method-specific components.

Despite this methodological progress, the influence of training data on the performance and generalization of DL-based RNA 3D structure quality predictors remains insufficiently characterized. Existing training datasets differ substantially in size, structural diversity, RNA length distributions, model generation protocols, and quality score distributions. Such differences can introduce dataset-specific biases that may strongly affect predictive behavior, particularly when predictors are applied to independent benchmarks or to models generated by different structure prediction pipelines. However, systematic studies that explicitly examine how alternative, nonnative training datasets affect the performance of DL-based quality assessment methods are lacking in the current literature.

In this work, we address this gap by presenting a comprehensive cross-training analysis of DL-based RNA 3D structure quality predictors. We characterize the existing training sets and an independently constructed new dataset, RNA3DQA, and analyze their structural and quality-related properties. Each predictor is retrained on nonnative datasets and evaluated on a common, independent test set derived from RNA-Puzzles benchmarks. We ensure that the benchmark test set contains neither PDB IDs from the training sets nor any Rfam families in common. This experimental design allows us to assess the relative impact of training data composition on predictor performance, robustness, and cross-dataset generalization. Our results demonstrate that the characteristics of training data play a decisive role in shaping the quality of reference-free RNA 3D structure assessment and can outweigh differences in predictive architectures. Based on these findings, we provide practical recommendations for dataset design and benchmarking strategies to improve the reliability and transferability of future DL-based predictors.

## Materials and methods

In this section, we compare the quantitative characteristics of three training datasets and the features of the benchmarked DL-based architectures. We then describe the independent benchmark set and the evaluation metrics used in this study.

### Training sets for RNA quality assessment

Three training datasets were used in this study: the ARES dataset [17], the lociPARSE dataset [18], and RNA3DQA, a new dataset constructed specifically for this work (Table 1). The RNA3DCNN training set is not available and, therefore, could not be included in the distributional analysis.

**Table 1:** Quantitative characteristics of the training sets.

	ARES	lociPARSE	RNA3DQA
Training units	10	1120	438
Training samples	10 000	41 440	10 095
Samples per unit	1000	37	3–29
Sequence length [nt]	21–47	10–197	10–182
RMSD range [Å]	1.69–16.82	0.28–161.15	0.48–58.75
Predictive methods	1	7	1

Training units are experimental reference structures, training samples are predicted structural models, samples per unit indicate the number of models per reference structure, and predictive methods denote the number of distinct methods used to generate the predicted 3D models. Sequence length denotes the range of reference sequence lengths in each dataset, while RMSD range indicates the minimum and maximum RMSD values between predicted models and their corresponding reference structures.

The ARES dataset [17] comprised 14 000 RNA 3D structural decoys derived from 14 reference structures, divided into a training set of 10 000 structural models and a validation set of 4000 structural models. For each target, 1000 *in silico* models were generated using FARFAR2 [19], resulting in the remaining entries in the dataset. Their lengths ranged from 21 to 47 residues in the training set and from 19 to 28 residues in the validation set, indicating relatively small and structurally tractable RNAs. To assess potential redundancy within the ARES dataset, we performed two complementary analyses. First, we examined whether experimentally determined RNA structures present in the training and validation splits could be assigned to the same Rfam families. In the training set, only one experimentally determined structure was assigned to an Rfam family, specifically the Coronavirus 3’ stem-loop II-like motif family (RF00164), while the remaining nine experimental targets could not be mapped to any known Rfam families. In the validation set, only one experimentally determined structure was assigned to an Rfam family, corresponding to the Human immunodeficiency virus type 1 major splice donor family (RF01380). Importantly, these families are distinct, indicating no overlap between training and validation sets at the level of Rfam annotations. Second, we evaluated sequence-level redundancy using CD-HIT [20]. This analysis revealed a single cluster at 88.46% sequence identity, comprising two structures of viral RNA pseudoknots, 1L2X [21] and 2A43 [22]. This indicates that the dataset is largely nonredundant, with only a minimal degree of sequence similarity between a pair of entries. Finally, we assessed structural similarity within the ARES set by analyzing the distribution of RMSD values between *in silico* models and their corresponding targets. This analysis revealed that 99.5% of models exhibit RMSD in the range of 2–16 Å, indicating that the generated decoy structures span a broad spectrum of structural deviations [23].

The lociPARSE dataset [18] contains 51 723 RNA 3D structures, divided into a training set of 41 440 and a validation set of 10 323. The training data comprised 1 120 experimentally determined RNA reference structures, while the validation set included 279 experimental reference structures. All *in silico* models were generated using a diverse set of RNA 3D structure prediction methods (Table S1 in the Supplementary Materials). To assess redundancy and composition of the lociPARSE dataset, we first analyzed the distribution of reference structures across Rfam families [24]. The training set comprises structures assigned to 56 unique Rfam families, covering 23.2% of all reference structures. Within this subset, 29 families contain at least two structures (Figure S7 in the Supplementary Materials). The most abundant family is RF00005 (tRNA), represented by 103 structures, followed by RF00167 (Purine riboswitch aptamer) with 18 structures, and RF00177 (Bacterial small subunit ribosomal RNA) with 10 structures. Overall, *∼ *10% of the training set consists of tRNA structures from the RF00005 family. In the validation set (Figure S8 in the Supplementary Materials), structures are assigned to 30 unique Rfam families, covering 25.8% of all reference structures, with 11 families represented by at least two structures. As in the training set, RF00005 (tRNA) is the most frequent family, comprising 26 structures, followed by RF00167 with six structures. Importantly, the training and validation sets share 22 Rfam families, indicating substantial overlap at the level of structural families and suggesting that the dataset is not strictly partitioned with respect to RNA family classification. In addition, sequence-level redundancy was assessed using CD-HIT with an 85% sequence identity threshold, which identified 39% of structures as redundant. We further evaluated the quality of experimental reference structures in the training set. Among the 956 reference structures, 222 were determined by NMR spectroscopy, which introduces additional uncertainty in the reference coordinates compared with high-resolution crystallographic structures. The remaining 541 structures were solved by X-ray crystallography, 193 by Electron Microscopy, with a median resolution of 3.1 Å. Finally, we analyzed the distribution of RMSD values across *in silico* models. These values ranged from 0.28 to 160 Å, with a substantial fraction of high-deviation models. We also examined whether RMSD correlates with RNA length; however, no significant correlation was observed (Spearman’s rho = 0.11). The distribution of decoy models across sequence lengths and their corresponding RMSD values is presented in Fig. 1 (panels A and C). To improve visualization, the zoomed-in views restricted to models with RMSD *≤ *16 Å are shown in Figure S9 in the Supplementary Materials.

**Figure 1:**
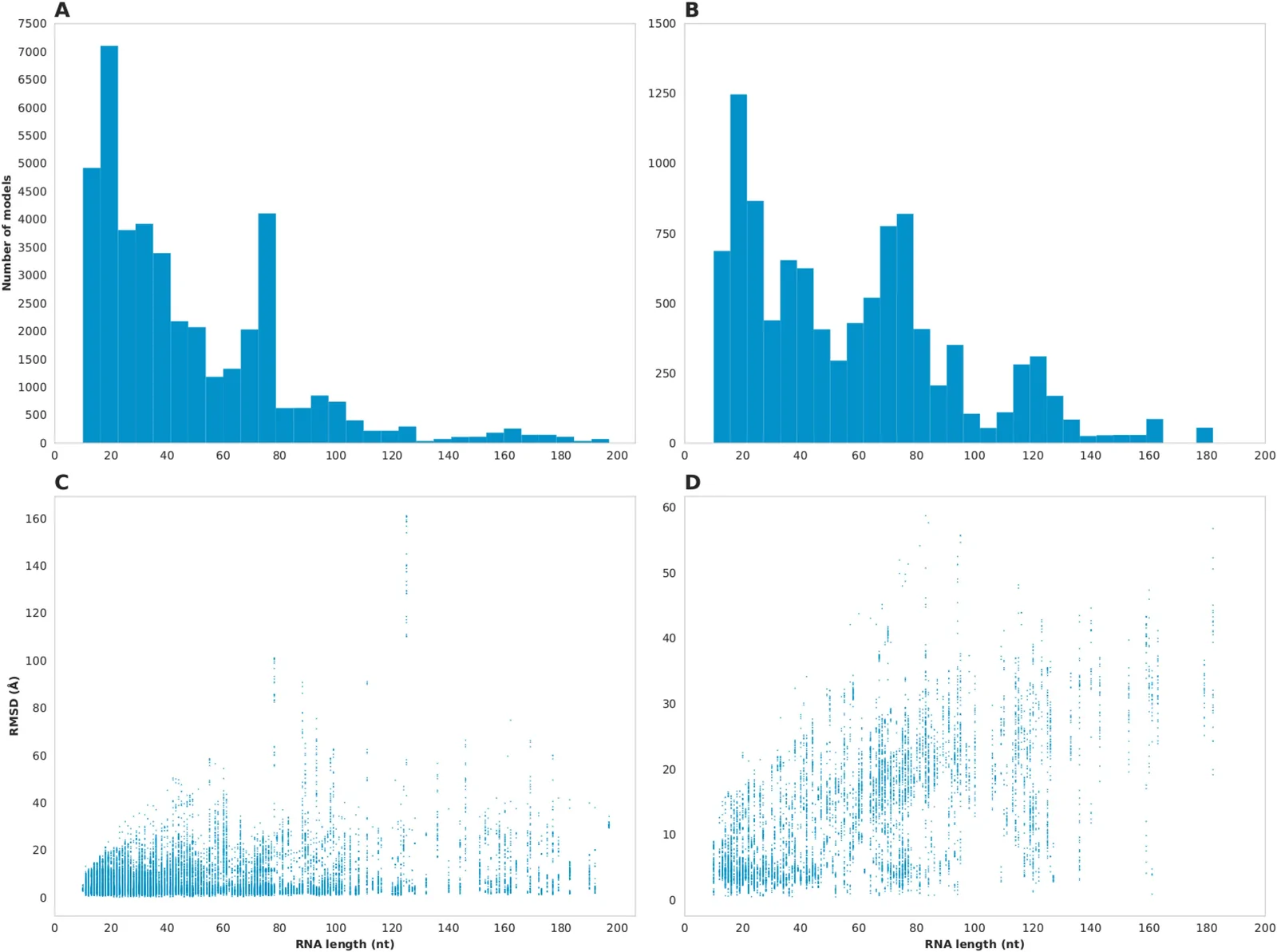
Quantitative characterization of two training datasets used in this study. Column 1 (Panels A and C) displays data for the lociPARSE dataset, while Column 2 (Panels B and D) represents the RNA3DQA dataset. The top row (Panels A and B) shows the distribution of decoy models across different RNA sequence lengths, where the peaks reflect the varying frequency of different target sizes in the prediction pools used to generate the decoys. The bottom row (Panels C and D) plots decoy RMSD against RNA sequence length, illustrating that decoy models of similar sequence lengths span a wide, diverse range of structural qualities (RMSD values) across all represented size scales.

The RNA3DQA dataset was constructed specifically for this study using version 3.306 of the BGSU nonredundant RNA 3D collection available through the RNAsolo2 database [25]. Starting from 3 837 representative RNA structures downloaded from RNAsolo2, we applied several filtering criteria to obtain a set of high-quality reference structures. Only structures determined by X-ray crystallography were considered. Specifically, we retained RNAs containing between 10 and 200 nucleotides, solved at a resolution of 3.5 Å or better, and exhibiting a predominantly structured architecture, defined as having at least as many paired as unpaired nucleotides. The resulting set of reference structures had a median resolution of 2.4 Å. Sequence-level redundancy was subsequently assessed using CD-HIT with an 85% sequence identity threshold [20], and redundant sequences were removed before dataset construction. To generate training and validation splits, we first characterized the filtered RNAs using RNAdistance-based secondary-structure similarity analysis [26]. The resulting structures were then clustered using k-means [27] (k = 25) in a 2D space defined by RNA length and median RMSD. Each cluster was partitioned according to a 6:2:2 ratio, preserving the distribution of structural complexity and model quality across the subsets. This procedure yielded 720 reference structures, of which 438 (60.8%) were assigned to the training set, 161 (22.3%) to the validation set, and 121 (16.8%) to the external test set. For each reference structure, *in silico* RNA 3D models were generated using RNAComposer [28]. The modeling pipeline extracted the RNA sequence and secondary structure from the reference using RNApdbee [29] and generated alternative secondary structures using multiple prediction tools: RNAshapes [30] and HotKnots [31] (five 2D structures generated by each tool), as well as RNAfold [26], RNAstructure [32], CONTRAfold [33], CentroidFold [34], ContextFold [35], and IPknot [36] (one 2D structure per tool). These secondary structures served as input for RNAComposer to generate corresponding 3D models. The accuracy of each model was evaluated by calculating RMSD relative to the reference structure [23]. Because not all prediction attempts were successful, the number of generated models varied across targets. The median number of models per reference structure was 25, with a range of 3 to 29. The final dataset comprised 10 095 entries for training and 4 321 entries for validation. To assess dataset redundancy, we analyzed the distribution of reference structures across Rfam families. As shown in Figures S10 and S11 (Supplementary Materials), both the training and validation sets were dominated by the RF00005 (tRNA) family, which accounted for *∼ *11% of reference structures. The next most abundant families were RF00001 (5S ribosomal RNA), representing 4.5% of structures, and RF00167 (Purine riboswitch aptamer), representing *∼ *1%. The training set contained structures assigned to 38 unique Rfam families, covering 29.6% of all reference structures, whereas the validation set contained 11 unique families, covering 24.7% of the reference structures. Although family-level leakage is present between both splits, these samples were intentionally retained as a control group to evaluate the model’s ability to memorize or distinguish known examples. We further analyzed the distribution of RMSD values across all *in silico* generated models. They ranged from 0.48 to 58.75 Å. We also investigated the relationship between RNA length (10-182 residues) and model accuracy. In contrast to the lociPARSE data, a moderate positive correlation was observed between structure size and RMSD (Spearman’s rho = 0.59), indicating that larger RNAs tend to be associated with less accurate models. The distribution of decoy models across sequence lengths and their corresponding RMSD values is presented in Fig. 1 (panels B and D). The distribution of decoy RMSD values across different length scales in panel D reflects populations of relatively easier (low RMSD) and more challenging (high RMSD) prediction targets. The zoomed-in views restricted to models with RMSD values not exceeding 16 Å are provided in Figure S9 in the Supplementary Materials.

For comparison, Fig. 2 illustrates the distributions of RMSD values across the lociPARSE, ARES, and RNA3DQA datasets. lociPARSE exhibits substantially more entries and a shift toward lower RMSD values. Its distribution is also more uniform, whereas ARES shows two pronounced peaks and RNA3DQA shows two less prominent peaks. These differences are consistent with the distinct compositions and preprocessing strategies used to construct the respective datasets.

**Figure 2:**
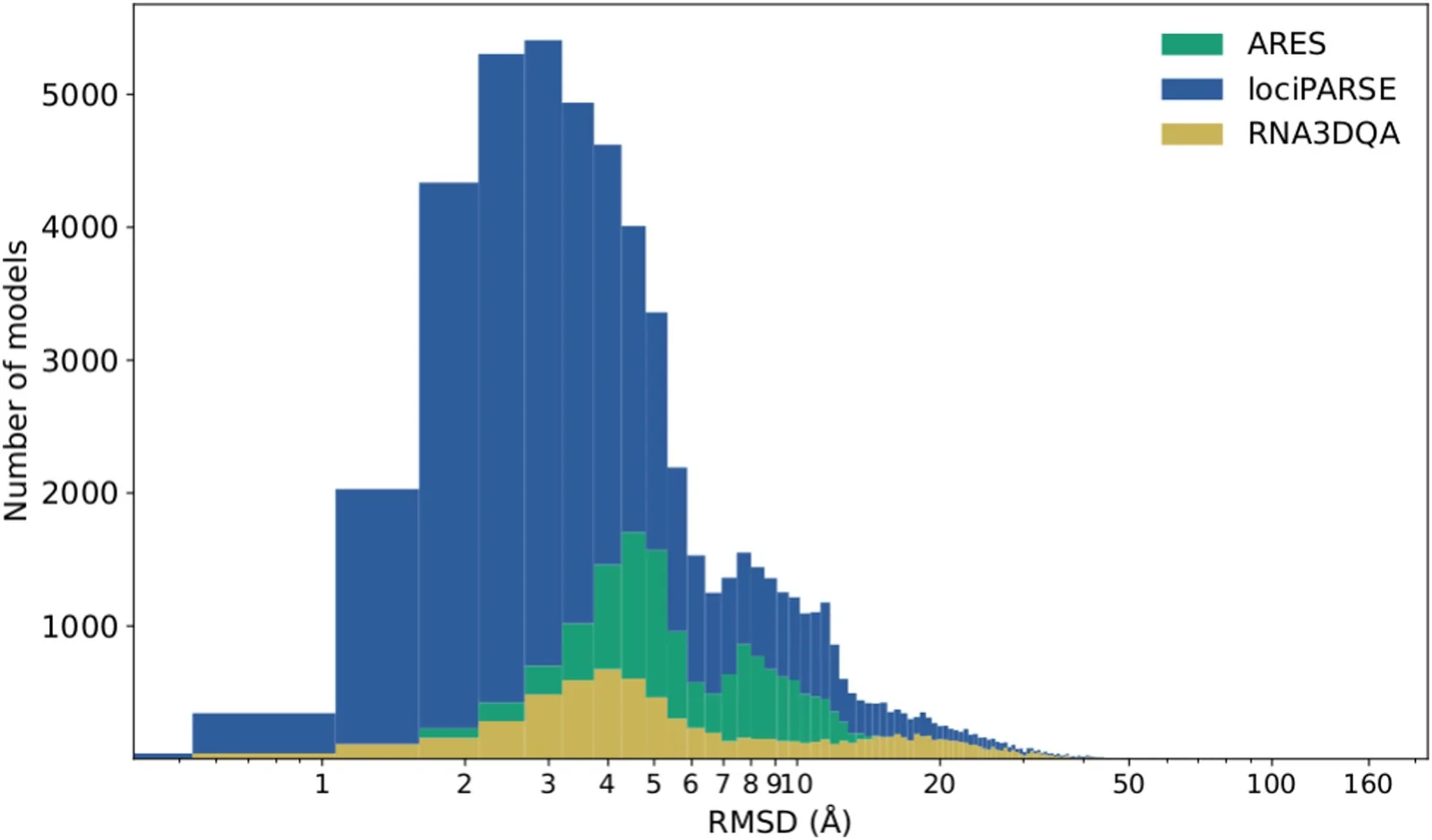
RMSD distribution in three training datasets used for RNA 3D structure quality assessment: ARES, lociPARSE, and RNA3DQA. The stacked histogram shows the number of structures in each RMSD interval.

### Deep learning models for predicting RNA 3D structure quality

In this study, we benchmarked three DL-based models for RNA 3D structure quality assessment, including ARES [17], lociPARSE [18], and RNA3DCNN [16], with key architectural features summarized in Table 2.

**Table 2:** Key features of the evaluated RNA 3D quality predictors.

	ARES	lociPARSE	RNA3DCNN
# parameters	18.1k	2.0 million	4.28 million
Training Code Availability	Available (also via Docker image)	Available (from repo history)	Unavailable (re-implemented in this study)
Training data availability	Public (10 000 decoys)	Public (41 440 decoys)	Unavailable
Software dependencies	Python 3.7, PyTorch 1.5.0, e3nn (custom)	Python 3.10, PyTorch 1.12.0	Python 3.10, Keras/PyTorch (our implementation)
Hardware requirements	NVIDIA GPU (Turing, e.g., RTX 2080 Ti; *≥ *12GB)	Modern NVIDIA GPU (e.g., H100; *≥ *16GB)	NVIDIA GPU (e.g., H100, RTX; *≥ *16GB)
Inference	Docker-based	Package-based (PyPI)	Script-based
CNN	RBF kernel (Gaussian)	Not applicable	3D image
Transformer	Not applicable	✓	Not applicable
GNN	✓	✓	Not applicable
Training data	PDB (RMSD labels)	PDB (IDDT labels)	Not available
Output	Global RMSD only	Local/global IDDT	Local/global RMSD
Code repository	https://zenodo.org/records/6893040	https://github.com/Bhattacharya-Lab/lociPARSE	https://github.com/lijunRNA/RNA3DCNN
Preprocessing	Expects a C/O/N-to-other-atoms ratio of *∼ *0.63. No preprocessing required.	Pre-inference feature extraction script.	Built-in inference script.

ARES. The architecture of ARES [17] is based on a graph convolutional neural network [37], which combines concepts from convolutional neural networks (CNNs) and graph neural networks (GNNs) by extending convolution operations to graph-structured representations of molecular systems. The model processes atoms represented as a point cloud with explicit spatial information, where each atom is connected to its 50 nearest neighbors. The authors employ a Gaussian Radial Basis Function (RBF) kernel [38] within the convolution operations, which mimics atomic electron-density fields and enables the network to learn distance-dependent interactions between neighboring atoms. These equivariant convolutions capture detailed geometric features by integrating two components: radial functions, which depend solely on Euclidean distances between atoms, and angular functions, which account for relative orientations of spatially proximate atoms. The model predicts RNA 3D structure quality by directly estimating the RMSD value of a given structural model. For practical usage, we provide a Docker image that facilitates training and prediction with ARES, as building the original software from source is challenging due to deprecated dependencies in the upstream repository. To enable GPU acceleration, an NVIDIA GPU with the sm_75 microarchitecture or older is required, along with PyTorch 1.5.0.

lociPARSE. The architecture of lociPARSE [18] is based on Invariant Point Attention (IPA), originally introduced in AlphaFold2 [39], which combines geometric attention mechanisms with message-passing operations over graph-structured representations of biomolecules. In this formulation, the model extends concepts from GNNs by incorporating invariant geometric transformations, enabling learning of spatial relationships independent of global orientation. The RNA structure is represented as a graph in which edges connect each nucleotide to its 20 nearest neighbors based on C4’ atom distances and discretized sequence separation. Nodes are encoded as one-hot representations of nucleobases, augmented with scalar features describing sequence distance from the first residue. lociPARSE operates on single-stranded RNA structures and predicts per-nucleotide quality scores as well as local atomic accuracy, with quality scores expressed in terms of predicted lDDT values, integrating both geometric and sequence-derived contextual information.

RNA3DCNN. The architecture of RNA3DCNN [16] is based on a 3D CNN, followed by two fully connected layers for final prediction. In this formulation, the predictive model operates on voxelized representations of RNA structures, where each structure is transformed into a set of 3D grids encoding electron density, mass density, and charge distribution within a bounded cubic volume. The network processes local structural environments using fixed-size 32*× *32*× *32 voxel subvolumes rather than the entire macromolecular structure, enabling focused analysis of local geometric features and efficient parallel computation. This design allows capturing spatial patterns in RNA structures through localized 3D convolutional filters applied to density-based representations. RNA3DCNN performs quality assessment by regressing the RMSD value of the RNA 3D structure.

Despite their architectural differences, the evaluated models differ substantially in their input representations and preprocessing requirements. ARES operates on structure-level representations and predicts a single RMSD value per RNA structure, whereas lociPARSE and RNA3DCNN use residue-level or local representations and require per-nucleotide or local scoring schemes. Although all models operate on RNA structures derived from the Protein Data Bank (PDB), their preprocessing pipelines differ. Due to the lack of reference structures, only PDB identifiers (ARES and lociPARSE) and, in some cases, chain identifiers (lociPARSE only) were originally provided. Therefore, all structures were retrieved from the PDB and transformed strictly according to the model-specific input pipelines provided by their respective authors.

### Cross-dataset training protocol

In this study, we applied a systematic cross-dataset training protocol in which all DL-based models were retrained on all available training datasets to evaluate their generalization across nonnative data distributions. Since ARES and lociPARSE provide complete training data and reproducible pipelines, their retraining was straightforward. In contrast, RNA3DCNN does not utilize a standardized training dataset and is distributed only as pretrained weights serialized in the HDF5 format; therefore, we reimplemented its architecture and training procedure based on the description in [16] and related documentation. In addition, we evaluated several available off-the-shelf models, including lociPARSE, ARES, and two RNA3DCNN variants. The first variant, RNA3DCNN_MD, was trained exclusively on decoys generated via molecular dynamics (MD). The second, RNA3DCNN_MDMC, was trained on a combined dataset of decoys generated using both MD and Monte Carlo (MC) methods, as provided in the original release.

Training was performed using dataset-specific optimization settings. The initial learning rates were set to *0.02* for ARES, $2 \times 10^{-5}$ for lociPARSE, and $2 \times 10^{-3}$ for RNA3DCNN. Following [16], a reduce-on-plateau learning rate schedule was applied to RNA3DCNN, halving the learning rate when no improvement in validation loss was observed for five consecutive epochs. After each reduction, the patience counter was reset. Training was terminated when the learning rate dropped below a dataset-specific threshold defined as 1% of its initial value.

### Benchmark set for independent evaluation

To obtain an unbiased assessment of model performance, we constructed an independent benchmark dataset consisting of RNA targets and their corresponding predictions, strictly disjoint from all training data. RNA-Puzzles [40–43] served as the primary source of entries, comprising 39 RNA targets with published RNA-Puzzles prediction and evaluation results available to date. To ensure independence between training and evaluation data, we excluded all RNA targets whose native 3D structures shared an Rfam family with any RNA structure present in the training sets. We also removed all associated predictions for these targets to eliminate any potential data leakage fully. This filtering procedure preserves the integrity of the benchmark, as it does not affect the trained models themselves but only reduces the evaluation set. The final benchmark dataset, named RNA-Puzzles-Bench, was constructed by considering RNA type, Rfam family membership, and PDB identifiers. It includes the following categories of RNA targets: RNA elements (three targets), viral elements (one target), ribozymes (11 targets), aptamers (two targets), and riboswitches (six targets), together with their corresponding RNA-Puzzles prediction and evaluation results. Each target defines a benchmark unit consisting of a single experimentally determined reference structure and a collection of computationally predicted models generated by different participating methods. Consequently, RNA-Puzzles-Bench comprises 23 benchmark units, one for each target, with the number of predicted models per unit ranging from 25 to 110. The reference RNA structures span 10 to 720 nucleotides in length. The RMSD values between predicted models and their corresponding reference structures range from 1.07 to 59.63 Å, while the lDDT values range from 0 to 0.89. Overall, the benchmark dataset contains 1546 predicted models.

### Evaluation protocols

To evaluate the performance of quality prediction methods in a meaningful way, we assess how well the predicted quality scores preserve the correct ranking of *in silico* RNA structural models within each benchmark unit. We compute the Spearman rank correlation coefficient between the reference ranking and the ranking induced by these predicted scores. Importantly, the reference rankings are constructed using the same quality measure as that predicted by the corresponding method: for ARES and RNA3DCNN, they are based on RMSD values computed with respect to the native structure using the Biopython library (Python 3) with explicit residue-level mapping to ensure consistent structural alignment, whereas for lociPARSE, they are based on lDDT values computed using OpenStructure v2.11 [44]. To aggregate performance across benchmark units, we introduce two scoring schemes:

- Top-3 scoring. For each benchmark unit from the RNA-Puzzles-Bench, only predictive models achieving a Spearman correlation *≥ * 0.5 are considered. Among them, the three best-performing predictive models are selected and awarded 3, 2, and 1 points for first-, second-, and third-place, respectively. The final score of a predictive model is obtained by summing the points accumulated across all benchmark units. Since RNA-Puzzles-Bench consists of 23 benchmark units and a maximum of 3 points can be obtained per unit, the total score ranges from 0 to 69, with higher values indicating better overall performance.
- Correlation scoring. Predictive models are assigned points directly based on their Spearman correlation coefficient: 5 points for *≥ * 0.9, 4 points for *≥ * 0.8, 3 points for *≥ * 0.7, 2 points for *≥ * 0.6, 1 point for *≥ * 0.5, and 0 points otherwise. The final score is obtained by summing points across all benchmark units and therefore ranges from 0 to 115. The maximum score of 115 corresponds to the case where a model achieves a Spearman correlation *≥ * 0.9 for all 23 benchmark units (i.e. 23*× *5=115).

For both scoring schemes, a baseline or uninformative predictor (e.g., random ranking achieving Spearman correlation < 0.5 across benchmark units) receives a score of 0. Conversely, the theoretical maximum scores (69 for Top-3 and 115 for Correlation scoring) represent an ideal predictor achieving near-perfect ranking (Spearman correlation *≥ * 0.9) across all 23 benchmark units. Given the inherent difficulty of reference-free RNA 3D structure quality assessment on nonredundant targets, these absolute scores reflect the overall challenging nature of the benchmark.

To assess the statistical reliability and significance of the reported Spearman rank correlations across individual benchmark units, we performed nonparametric bootstrapping with 1000 resamples to estimate 95% confidence intervals (CIs). For each bootstrap replication, predicted structural models within a benchmark unit were sampled with replacement, and the Spearman correlation was recomputed. Furthermore, to determine whether differences in ranking errors between model configurations are statistically significant, we employed the one-sided paired Wilcoxon signed-rank test [45]. For each structural model, the rank error is defined as the absolute difference between its predicted rank and its ground-truth reference rank (induced by lDDT or RMSD):


(1) \begin{align*}& \textrm{Rank Error} = |\textrm{Predicted Rank} - \textrm{Reference Rank}|\end{align*}

The paired tests were evaluated across the ensemble of predicted structures for each of the 23 benchmark units independently.

### Computational resources

Training and evaluation were performed on two Linux workstations. RNA3DCNN and lociPARSE were run on a workstation equipped with an AMD EPYC 9334 CPU (64 cores, 3.90 GHz), 770 GB DDR4 RAM, and an NVIDIA H100 GPU (96 GB), operating under Rocky Linux 9.7 (Blue Onyx). ARES was evaluated on a separate workstation due to GPU-related limitations of its e3nn implementation, equipped with an Intel Core i7-8700 CPU (12 cores, 3.20 GHz), 16 GB DDR4 RAM, and an NVIDIA GeForce RTX 2080 Ti GPU (12 GB), operating under Ubuntu 22.04.2 LTS. Specifically, the legacy software dependencies of ARES (including PyTorch 1.5.0) are incompatible with the newer NVIDIA Hopper architecture of the H100 GPU, necessitating the use of the Turing-based RTX 2080 Ti. We confirmed that this hardware differentiation affects only the training and inference throughput (execution time) and has no impact on the predictions or model ranking metrics.

## Results of cross-training experiment

To investigate the impact of training data on DL-based RNA 3D structure quality prediction, we benchmarked 13 model configurations representing different architectures and training strategies. These comprised four off-the-shelf models (ARES, lociPARSE, RNA3DCNN_MD, and RNA3DCNN_MDMC); two retrained models, one each for ARES and lociPARSE; four models trained on nonnative datasets—two lociPARSE models trained on the ARES and RNA3DQA datasets, respectively, and two ARES models trained on the lociPARSE and RNA3DQA datasets, respectively; and three RNA3DCNN models trained on all datasets considered. We also evaluated retrained models to verify that the published datasets, source code, and hyperparameter settings are sufficient to reproduce the performance of the original off-the-shelf models.

The evaluation was based on the two quality metrics predicted by the assessed DL-based models, RMSD and lDDT. Accordingly, predicted RMSD values were obtained for ARES and RNA3DCNN, whereas predicted lDDT values were obtained for lociPARSE. Table 3 summarizes the ranges of the predicted scores together with their median deviations from the corresponding reference values, where the reference RMSD and lDDT were computed by comparing the RNA-Puzzles-Bench models with their target structures.

**Table 3:** Predicted quality metric ranges and median deviations on RNA-Puzzles-Bench.

Model	Training set	Metric	
(metric)		Range	Median *Δ *
ARES (RMSD)	ARES	5.30–14.54	6.24
	ARES (retrained)	4.04–14.54	5.50
	lociPARSE	1.42–28.27	9.85
	RNA3DQA	1.40–37.23	4.02
lociPARSE (lDDT)	ARES	0.32–0.73	0.10
	lociPARSE	0.43–0.82	0.14
	lociPARSE (retrained)	0.40–0.82	0.14
	RNA3DQA	0.25–0.83	0.08
RNA3DCNN (RMSD)	ARES	1.35–32.93	8.77
	lociPARSE	0.52–7.30	12.69
	RNA3DCNN_MD	8.57–172.00	20.66
	RNA3DCNN_MDMC	19.00–348.80	34.87
	RNA3DQA	2.42–33.30	8.11

The impact of training data on model performance was evaluated using two scoring systems: *Top-3 scoring* and *Correlation scoring*. The results of the first scheme are summarized in Table 4. Under this scheme, the lociPARSE dataset proved to be the most effective overall, yielding the highest-scoring combination (19 points across seven top-three finishes: 5 *× * first and 2 *× * second place) and enabling the ARES architecture to achieve its peak performance (6 points; fourth place). The RNA3DQA dataset showed competitive performance when paired with the lociPARSE architecture, achieving a second-place finish with 17 points across nine top-three finishes (3 *× * first, 2 *× * second, and 4 *× * third place), although no improvement was observed for the corresponding ARES-based configuration. Furthermore, in several high Spearman-correlation challenges, the RNA3DQA-trained model was consistently ranked second behind the off-the-shelf lociPARSE model, which explains its overall reduced contribution to the top-score aggregation. The retrained lociPARSE model secured third place with 16 points across eight top-three finishes (2 *× * first, 4 *× * second, and 2 *× * third place), whereas models trained on the ARES dataset and RNA3DCNN_MDMC achieved substantially lower scores, placing their respective architectures in the lower half of the ranking.

**Table 4:** Model rankings under the *Top-3 scoring* scheme (score range: 0–69).

Rank	Model	Training set	Score
1	lociPARSE	lociPARSE	19
2	lociPARSE	RNA3DQA	17
3	lociPARSE	lociPARSE (retrained)	16
4	ARES	lociPARSE	6
5	ARES	ARES	5
6	RNA3DCNN	RNA3DCNN_MDMC	3
7	lociPARSE	ARES	2
7	ARES	ARES (retrained)	2
9	RNA3DCNN	RNA3DCNN_MD	1
-	ARES	RNA3DQA	0
-	RNA3DCNN	RNA3DQA	0
-	RNA3DCNN	lociPARSE	0
-	RNA3DCNN	ARES	0

Table 5 presents the ranking of the evaluated models based on the *Correlation scoring* scheme. The results show a broadly consistent pattern with the ranking obtained under the *Top-3 scoring* scheme (Table 4). The lociPARSE architecture occupies the top positions across most configurations. Notably, the RNA3DQA-trained model achieves the highest score (27 points), followed by the retrained lociPARSE model (26 points) and the original lociPARSE model (25 points), suggesting that this architecture benefits from training on diverse datasets. ARES shows moderate performance, ranking fourth and fifth, with the lociPARSE training set (10 points) again outperforming its native dataset (8 points). RNA3DCNN consistently ranks near the bottom, with no configuration exceeding 4 points; its variants trained on nonnative datasets do not achieve any score, indicating limited benefit from cross-dataset training for this architecture. The relatively low absolute scores compared with the maximum possible value reflect the difficulty of the benchmark. However, the relative ordering of architectures—lociPARSE, followed by ARES, and then RNA3DCNN—remains stable across both evaluation schemes, suggesting that architectural design plays a major role in performance, particularly when combined with high-quality training datasets such as RNA3DQA and lociPARSE.

**Table 5:** Model rankings under the *Correlation scoring* scheme (score range: 0–115).

Rank	Model	Training set	Score
1	lociPARSE	RNA3DQA	27
2	lociPARSE	lociPARSE (retrained)	26
3	lociPARSE	lociPARSE	25
4	ARES	lociPARSE	10
5	ARES	ARES	8
6	RNA3DCNN	RNA3DCNN_MD	4
7	ARES	ARES (retrained)	3
8	RNA3DCNN	RNA3DCNN_MDMC	2
8	lociPARSE	ARES	2
10	ARES	RNA3DQA	1
-	RNA3DCNN	RNA3DQA	0
-	RNA3DCNN	lociPARSE	0
-	RNA3DCNN	ARES	0

While the aggregate benchmark scores for top-performing configurations such as lociPARSE show overall parity between native and cross-trained variants (e.g., 26 versus 27 points in Table 5), the practical benefits of cross-training are target-specific, yielding substantial improvements on individual benchmark units and for architectures with lower baseline performance (such as ARES).

To verify the ranking performance gains at a granular, target-specific level, we analyzed the individual Spearman correlations, bootstrapped 95% CIs, and associated *P*-values across all 23 benchmark units. Complete statistical validation tables for the ARES, lociPARSE, and RNA3DCNN architectures under all evaluated training configurations are provided in Supplementary Tables S2, S3, and S4, respectively. For the top-performing lociPARSE architecture (Supplementary Table S3), training on the RNA3DQA dataset consistently yields high and statistically significant correlations (*P <0.05*) across almost all target structures. Crucially, on challenging targets where the native lociPARSE model failed to produce meaningful rankings (such as PZ15, where the native model correlation is near-zero and statistically nonsignificant, $r_{s} = -0.13, P =0.1970$), retraining on RNA3DQA transforms the predictor into a statistically significant ranker ($r_{s} = 0.27, P =0.0065$). This target-by-target analysis confirms that the expanded structural diversity of RNA3DQA provides a highly reliable signal across distinct RNA topologies.

To identify a statistically superior ranking solution, we conducted a detailed comparative analysis of absolute rank errors using the paired Wilcoxon signed-rank test [45] on the two leading configurations (original lociPARSE versus RNA3DQA-trained lociPARSE). Our results (Supplementary Table S5) reveal a symmetry: each model performs significantly better on exactly six of the 23 test cases, with no significant difference observed in the remaining 11 contests. This balanced performance profile suggests that the two models have complementary strengths, excelling on different problem instances based on their inherent biases and training paradigms. Notably, RNA3DQA-trained lociPARSE dominates on larger and more complex puzzles (particularly PZ15 and PZ17), while the original lociPARSE remains superior on smaller or differently structured cases. These findings motivate the development of an ensemble approach that leverages both models’ respective strengths.

Furthermore, to assess the impact of random weight initialization on model stability, we evaluated ranking performance across four independent random seeds for lociPARSE and RNA3DCNN retrained on the RNA3DQA dataset. Because ARES has substantial computational overhead and high memory requirements, we focused multi-seed evaluations on these two architectures. As shown in Supplementary Figures S15 and S16, the target-specific Spearman correlation distributions remain highly consistent across training runs, confirming that the observed performance on RNA3DQA is robust and reproducible.

### Training data impact on model performance

To assess the effect of training data on predictive performance, we evaluated 13 model configurations (DL-based architectures combined with different training sets) on the RNA-Puzzles-Bench dataset. Figure 3 summarizes the results aggregated over 23 benchmark units. Overall, the effect of cross-dataset training is strongly architecture-dependent. The lociPARSE architecture shows the most consistent improvements when trained on nonnative datasets, with RNA3DQA and ARES providing performance gains across multiple aggregation schemes, particularly for median- and maximum-based evaluations. In contrast, the ARES architecture exhibits more mixed behavior, with improvements limited to specific training configurations (most notably lociPARSE-trained ARES under maximum aggregation), while other cross-training setups result in marginal or negative changes. RNA3DCNN is the most stable but least adaptable architecture, showing consistently negative or negligible changes across all training datasets and aggregation strategies. Notably, no cross-training configuration leads to a consistent performance gain for RNA3DCNN, indicating limited benefit from nonnative training data for this model class. Across all architectures, the strongest positive effects are observed under maximum-based aggregation, where cross-training on lociPARSE or RNA3DQA occasionally yields substantial gains, whereas mean- and minimum-based aggregations tend to amplify performance degradation. This suggests that improvements induced by alternative training datasets are often driven by a subset of high-performing cases rather than uniform improvements across all targets.

**Figure 3:**
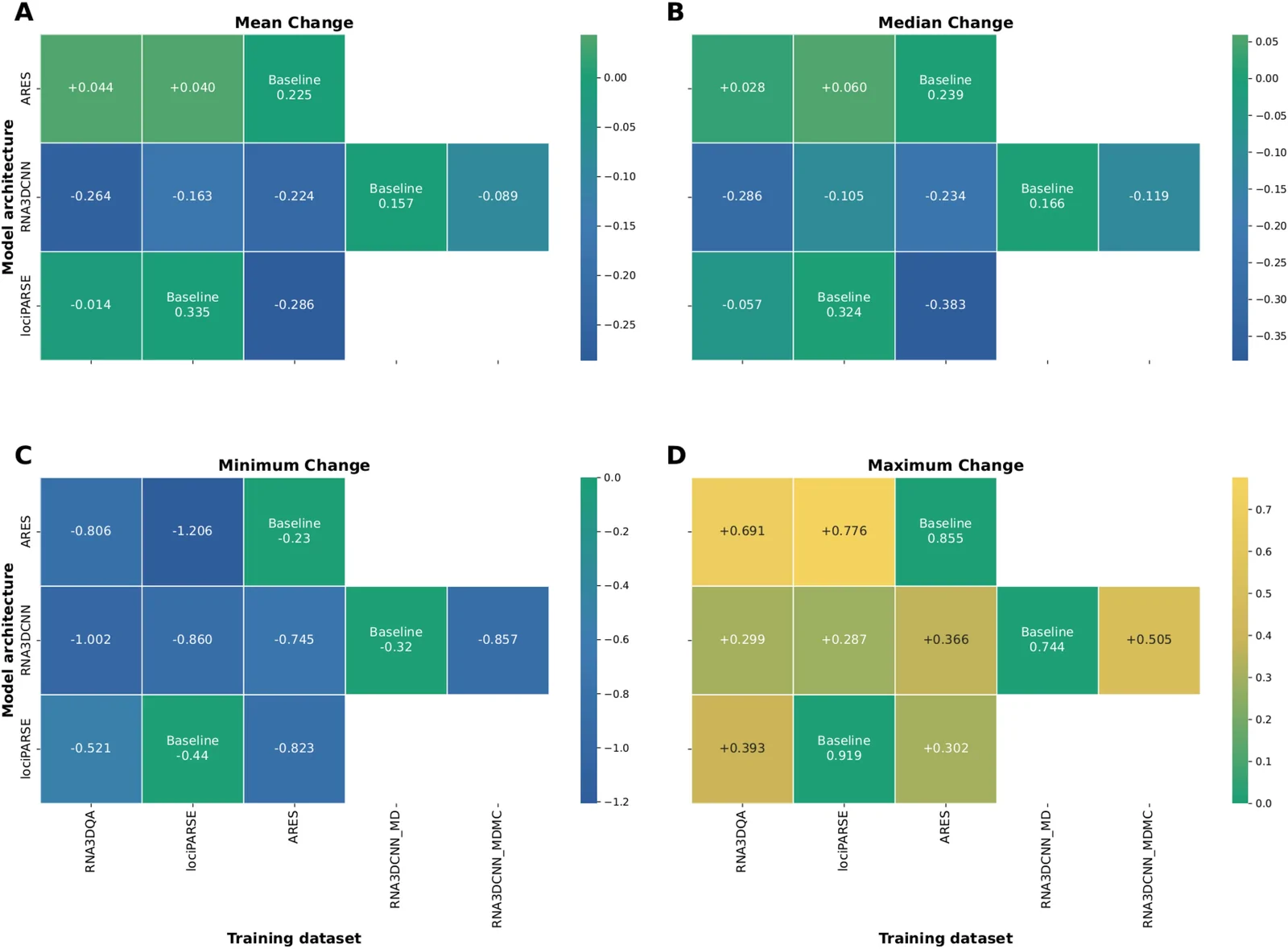
Changes in predictive performance on RNA-Puzzles-Bench across model architectures and training datasets. Performance is aggregated using (A) mean, (B) median, (C) minimum, and (D) maximum Spearman rank correlation coefficients over 23 benchmark units. The Y-axis indicates the evaluated model architecture, while the X-axis denotes the training dataset. For each architecture, the cell representing its native training set is designated as the reference point (”Baseline” followed by its absolute Spearman correlation value). All other cells in the same row display the relative change (delta) from this baseline ($\Delta \rho = \rho _{\textrm{retrained}} - \rho _{\textrm{baseline}}$). The positive values indicate a performance improvement after training on a nonnative dataset, while the negative values represent performance degradation. The empty cells represent configurations that could not be evaluated due to the unavailability of the native RNA3DCNN training pipeline.

### Cross-dataset generalization

We evaluated cross-dataset generalization to assess how model-specific input requirements and preprocessing pipelines influence the computation of quality scores across different RNA structure representations. The parameters of the fitted normal distributions—mean and standard deviation (SD)—of the Spearman correlation coefficients, used as a metric of prediction stability across the benchmark, are reported in Table 6. Figures S5–S6 in the Supplementary Materials show the distributions of Spearman correlation coefficients for each benchmark unit (puzzle) and each evaluated configuration. RNA3DCNN exhibits the lowest standard deviations overall (*≤ * 0.262), indicating a consistently tight score distribution across training sources, albeit with low or negative mean values. In contrast, lociPARSE shows the highest variance, with standard deviations around 0.400 across its best training sets. ARES is most sensitive to training data: when trained on RNA3DQA, it achieves its highest stability and accuracy (mean: 0.269, SD: 0.184), whereas training on lociPARSE substantially increases its variability (SD: 0.311).

**Table 6:** Spearman correlation score distributions on RNA-Puzzles-Bench.

Model	Training set	Mean	SD
ARES	ARES	0.225	0.285
	ARES_retrained	−0.003	0.307
	lociPARSE	0.265	0.311
	RNA3DQA	0.269	0.184
lociPARSE	ARES	0.050	0.371
	lociPARSE	0.335	0.391
	lociPARSE_retrained	0.326	0.402
	RNA3DQA	0.322	0.410
RNA3DCNN	ARES	−0.065	0.153
	lociPARSE	−0.006	0.246
	RNA3DCNN_MD	0.157	0.262
	RNA3DCNN_MDMC	0.068	0.222
	RNA3DQA	−0.098	0.182

The comparison of all three architectures, evaluated in off-the-shelf and RNA3DQA-retrained variants (Figures S1–S4 in the Supplementary Materials), does not identify a single best model across RNA types. For viral elements, the RNA3DQA-retrained lociPARSE performs similarly to its baseline. In ribozymes, retraining yields a positive gain in one of four benchmark units, but negative gains in the remaining ones, where lociPARSE previously showed high correlation. ARES benefits most from RNA3DQA training, with clear gains in one aptamer and one riboswitch unit, and shows the most stable behavior across ribozymes. The original RNA3DCNN model (without retraining) achieves the best performance in three benchmark units (one riboswitch and two ribozymes). Overall, ribozymes are the most challenging category across all evaluation protocols.

In individual rankings, lociPARSE leads with first place in 10 benchmark units and second in six, followed by ARES (eight first places, six second places), while RNA3DCNN achieves first place in only one unit. These rankings reflect the best-performing variant of each model across off-the-shelf and RNA3DQA-trained configurations.

The performance discrepancy of ARES across training datasets (Tables 6 and 5) is driven by the stability of RNA3DQA rankings. Its lower variance yields more consistent and reproducible predictions, whereas higher-variance training sets increase both the chance of isolated high scores and the risk of severe failures, leading to less predictable overall performance.

Findings from our cross-training analysis point to two factors for future training dataset design. First, existing datasets exhibit a significant imbalance—they are dominated by a few overrepresented families (such as tRNA, which accounts for *∼ *10% of structures) and consequently feature a limited variety of common secondary motifs (e.g., stems and simple hairpins). Hence, to reduce the predictive model’s bias toward simple, canonical geometries, intentionally oversampling underrepresented but structurally significant motifs (e.g., complex internal loops, multi-way junctions, and noncanonical base pairings) is recommended. Second, the performance shifts observed when evaluating models on nonredundant targets emphasize that strictly splitting training and validation data by RNA family or structural fold is critical to prevent data leakage and ensure true topological generalization.

### Case study: cross-training effect for benchmark unit PZ37

As a case study, we selected benchmark unit PZ37 from RNA-Puzzles-Bench, which illustrates the improvement in quality assessment achieved by the lociPARSE architecture when trained on the RNA3DQA dataset.

PZ37 corresponds to an NAD^+^-II riboswitch comprising 61 nucleotides [46]. This benchmark unit includes 90 RNA 3D models, with RMSD values ranging from 5.4 to 32 Å (lDDT scores spanning from 0.204 to 0.631). Many models differ only slightly, with 37 falling within the 12–16 Å RMSD range. To quantitatively evaluate how different training datasets influence ranking precision across structural quality regimes, we calculated the absolute rank error for each predicted model as defined in Equation 1, where the reference ranking is derived from the ground-truth lDDT scores relative to the native structure. To evaluate performance across structural quality regimes, the 90 predicted models were divided into three equal tiers of 30 structures based on reference lDDT values: high-, medium-, and low-quality. Figure 4 presents the distribution of absolute rank errors for PZ37 across four lociPARSE training configurations. Although the overall increase in Spearman correlation is substantial (*Δ * = 0.37, rising from 0.31 for the original model to 0.68 when trained on RNA3DQA), the rank error distribution offers a much clearer view of prediction stability. The RNA3DQA-trained lociPARSE model consistently achieves lower median rank errors and reduced variance across all three model groups compared with its off-the-shelf counterpart and other retrained variants. Crucially, it suppresses severe rank misassignments, preventing low-quality decoy structures from being falsely assigned high ranks.

**Figure 4:**
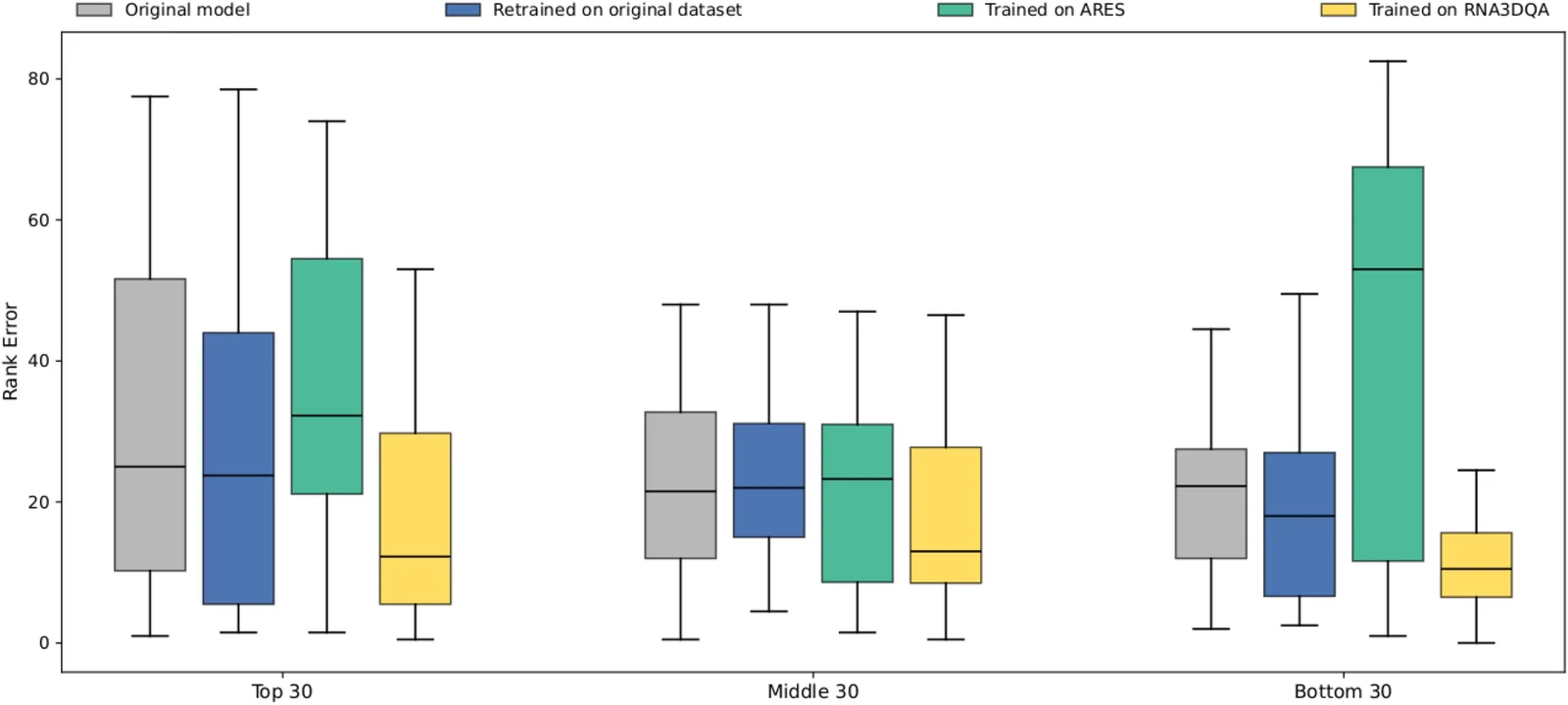
Distribution of absolute rank errors for benchmark unit PZ37 across lociPARSE training setups, stratified into three model quality tiers.

The ranking performance gains observed for PZ37 reflect a broader systemic trend. Global absolute rank error distributions evaluated across the entire RNA-Puzzles-Bench dataset for the lociPARSE, ARES, and RNA3DCNN architectures are provided in Supplementary Figures S12–S14, confirming that training on diverse datasets may improve ranking precision across diverse structural folds.

## Conclusions

In this work, we investigate how training data influence the performance and generalization of DL-based RNA 3D structure quality assessment models by systematically evaluating different combinations of model architectures and training datasets. To enable this study, we introduce a new dataset, RNA3DQA, comprising 720 high-resolution (*≤ *3.5Å) experimentally determined RNA 3D structures and over 18k corresponding RNA 3D models generated by RNAComposer. We systematically evaluated the generalization ability of all combinations of three DL-based architectures and three training datasets. Among the trained models, six predict RMSD-based scores and three predict lDDT-based scores. We benchmarked all models on the RNA-Puzzles-Bench dataset, consisting of 23 benchmark units. For each RNA 3D model, a quality score was predicted across all benchmark units, and Spearman correlation coefficients were computed per unit based on the agreement between predicted and reference rankings. This allowed assessment of both per-unit ranking quality and overall generalization performance.

The results show that accurate RNA 3D structure quality assessment remains challenging. Existing approaches often fail to reliably distinguish native-like structures among diverse *in silico* predictions. Both training data and model architecture strongly influence performance and robustness. Notably, models trained on external competitive datasets can outperform those trained on their original datasets, and the best-performing model across both evaluation protocols uses IPA and was trained on the RNA3DQA dataset.

These findings highlight that dataset design can be more critical than architectural choices. RNA structural data are scarce and highly redundant, making dataset construction a key challenge. Effective training data should cover a broad and balanced spectrum of structural quality using diverse prediction methods, enabling learning beyond method-specific artifacts. Multiple complementary metrics (e.g., RMSD, INF, and lDDT) should be used to capture both global and local structural features relevant to function. Underrepresented but structurally important motifs should be explicitly oversampled to reduce bias toward common elements such as helices and hairpins. In addition, splitting data by RNA family or fold is essential to prevent leakage and improve generalization to unseen topologies. Physics-based augmentation may further increase training diversity without compromising independence between training and test sets.

Our results suggest that DL-based RNA 3D structure quality assessment methods should be evaluated not only on native training datasets, but also across independent benchmark and training data, as cross-dataset testing provides a more reliable assessment of robustness and generalization.

**Key Points**

- Training data properties strongly influence the performance of deep learning–based RNA 3D structure quality predictors.
- Cross-training reveals that dataset composition can have a greater impact on prediction accuracy than model architecture.
- Reliable reference-free identification of native-like RNA 3D structures remains a major challenge.

## Supplementary Material

Adamczyk_supplementary_bbag630

## Data Availability

All datasets, trained models, and analysis scripts supporting this study are available in the RNA3DQA repository at Zenodo (https://doi.org/10.5281/zenodo.22712263). The repository includes redundancy-checked sequence data; prepared training datasets for lDDT and RMSD metrics; standardized RNA-Puzzles benchmark data; trained model weights for ARES, lociPARSE, and RNA3DCNN; and the inference pipeline for RNA3DCNN.
