# Supplementary material for: Impact of training data on deep learning–based RNA 3D structure quality assessment
Source: Brief Bioinform. 2026 Nov 9;27(6):bbag630. doi: 10.1093/bib/bbag630 (PMC13747016; doi:10.1093/bib/bbag630)

Supplementary information

Impact of training data on deep learning-based RNA 3D structure quality assessment

Bartosz Adamczyk[1,2], Marta Szachniuk[1,2], Maciej Antczak[1,2]

[1]Institute of Computing Science, Poznan University of Technology,
ul. Jacka Rychlewskiego 1, 61-131, Poznan, Poland
[2]Institute of Bioorganic Chemistry, Polish Academy of Sciences,
Noskowskiego 12/14, 61-704, Poznan, Poland

Table S1: Predictive methods used for the construction of the lociPARSE dataset and the corresponding numbers of 3D models generated per reference RNA structure.

**Method category**	**Method**	**# Models**
Deep learning-based	DeepFoldRNA	6
	trRosettaRNA	10
	RoseTTAFoldNA	1
	RhoFold	1
	DRfold	6
Physics- and/or knowledge-based	SimRNA	1
	PyRosetta perturbation	12
	Total:	**37**

Table S2: Statistical validation of ARES ranking performance across RNA-Puzzles-Bench targets under three training setups. The table presents the original Spearman rank correlation (r_s), 95% bootstrapped confidence intervals (CIs) from 1,000 resamples, and the associated p-values, comparing the ARES architecture trained on the original ARES, lociPARSE, and RNA3DQA datasets.

**Puzzle**	**Trained on ARES**			**Trained on lociPARSE**			**Trained on RNA3DQA**		
	r_s	**95% CI**	p**-value**	r_s	**95% CI**	p**-value**	r_s	**95% CI**	p**-value**
PZ5	-0.43	$[-0.75, 0.00]$	0.0334	0.10	$[-0.34, 0.49]$	0.6186	-0.41	$[-0.72, 0.01]$	0.0396
PZ6	-0.47	$[-0.79, -0.11]$	0.0049	0.16	$[-0.19, 0.47]$	0.3674	-0.78	$[-0.92, -0.53]$	0.0000
PZ7	-0.20	$[-0.48, 0.09]$	0.1524	0.50	$[0.30, 0.65]$	0.0001	0.22	$[-0.08, 0.51]$	0.1179
PZ9	-0.70	$[-0.79, -0.49]$	0.0000	-0.50	$[-0.67, -0.22]$	0.0026	-0.30	$[-0.57, 0.03]$	0.0822
PZ14Bound	-0.34	$[-0.57, -0.10]$	0.0057	-0.56	$[-0.71, -0.36]$	0.0000	-0.37	$[-0.59, -0.12]$	0.0027
PZ14Free	-0.37	$[-0.62, -0.08]$	0.0072	-0.66	$[-0.79, -0.44]$	0.0000	-0.41	$[-0.63, -0.15]$	0.0024
PZ15	0.40	$[0.20, 0.55]$	0.0000	0.35	$[0.19, 0.48]$	0.0002	0.36	$[0.15, 0.54]$	0.0002
PZ15noend	0.25	$[-0.05, 0.47]$	0.0336	0.16	$[-0.10, 0.37]$	0.1778	0.24	$[-0.05, 0.48]$	0.0413
PZ15rec	0.25	$[-0.06, 0.47]$	0.0340	0.20	$[-0.04, 0.39]$	0.0839	0.25	$[-0.03, 0.49]$	0.0353
PZ16a	-0.01	$[-0.25, 0.22]$	0.9174	0.41	$[0.19, 0.60]$	0.0000	-0.25	$[-0.44, -0.03]$	0.0186
PZ16b	0.10	$[-0.12, 0.32]$	0.2932	-0.77	$[-0.84, -0.67]$	0.0000	-0.57	$[-0.67, -0.44]$	0.0000
PZ17	-0.24	$[-0.42, -0.04]$	0.0110	-0.31	$[-0.47, -0.12]$	0.0008	-0.48	$[-0.61, -0.32]$	0.0000
PZ18	-0.36	$[-0.55, -0.12]$	0.0016	-0.58	$[-0.72, -0.40]$	0.0000	-0.41	$[-0.61, -0.18]$	0.0002
PZ19	-0.35	$[-0.56, -0.08]$	0.0037	-0.09	$[-0.33, 0.18]$	0.4899	-0.40	$[-0.58, -0.17]$	0.0007
PZ20	0.31	$[0.06, 0.53]$	0.0118	0.18	$[-0.07, 0.41]$	0.1422	0.17	$[-0.12, 0.41]$	0.1883
PZ22	0.66	$[0.46, 0.79]$	0.0000	0.29	$[-0.03, 0.54]$	0.0386	0.41	$[0.14, 0.65]$	0.0028
PZ23	0.41	$[0.05, 0.68]$	0.0137	0.18	$[-0.16, 0.52]$	0.2923	-0.28	$[-0.57, 0.05]$	0.1079
PZ30	0.55	$[0.19, 0.77]$	0.0008	0.15	$[-0.24, 0.49]$	0.4017	-0.33	$[-0.61, -0.01]$	0.0537
PZ32	-0.26	$[-0.43, -0.06]$	0.0074	-0.52	$[-0.64, -0.36]$	0.0000	-0.40	$[-0.54, -0.23]$	0.0000
PZ34	-0.55	$[-0.70, -0.37]$	0.0000	0.22	$[0.02, 0.40]$	0.0282	-0.20	$[-0.38, 0.01]$	0.0465
PZ36	-0.52	$[-0.73, -0.22]$	0.0008	-0.75	$[-0.87, -0.56]$	0.0000	0.12	$[-0.21, 0.43]$	0.4575
PZ37	0.21	$[0.01, 0.42]$	0.0460	-0.46	$[-0.61, -0.30]$	0.0000	-0.18	$[-0.39, 0.03]$	0.0825
PZ38	0.01	$[-0.20, 0.22]$	0.9270	-0.24	$[-0.46, -0.02]$	0.0252	-0.25	$[-0.48, -0.02]$	0.0201

Table S3: Statistical validation of lociPARSE ranking performance across RNA-Puzzles-Bench targets under three training setups. The table presents the original Spearman rank correlation (r_s), 95% bootstrapped confidence intervals (CIs) from 1,000 resamples, and the associated p-values, comparing the lociPARSE architecture trained on the original lociPARSE, ARES, and RNA3DQA datasets.

**Puzzle**	**Trained on lociPARSE**			**Trained on ARES**			**Trained on RNA3DQA**		
	r_s	**95% CI**	p**-value**	r_s	**95% CI**	p**-value**	r_s	**95% CI**	p**-value**
PZ5	0.26	$[-0.23, 0.68]$	0.2099	-0.12	$[-0.54, 0.36]$	0.5620	-0.26	$[-0.77, 0.21]$	0.2076
PZ6	0.60	$[0.28, 0.79]$	0.0002	0.58	$[0.31, 0.72]$	0.0004	0.47	$[0.07, 0.71]$	0.0055
PZ7	-0.05	$[-0.33, 0.23]$	0.7422	0.26	$[-0.05, 0.52]$	0.0674	-0.15	$[-0.41, 0.13]$	0.2899
PZ9	0.71	$[0.44, 0.86]$	0.0000	0.40	$[0.09, 0.65]$	0.0199	0.65	$[0.41, 0.79]$	0.0000
PZ14Bound	0.77	$[0.64, 0.85]$	0.0000	0.39	$[0.17, 0.57]$	0.0015	0.60	$[0.41, 0.72]$	0.0000
PZ14Free	0.88	$[0.79, 0.93]$	0.0000	0.43	$[0.21, 0.66]$	0.0016	0.80	$[0.68, 0.88]$	0.0000
PZ15	-0.13	$[-0.29, 0.08]$	0.1970	-0.57	$[-0.66, -0.45]$	0.0000	0.27	$[0.06, 0.47]$	0.0065
PZ15noend	-0.01	$[-0.26, 0.25]$	0.9102	-0.44	$[-0.60, -0.23]$	0.0001	0.23	$[-0.01, 0.47]$	0.0441
PZ15rec	-0.03	$[-0.26, 0.23]$	0.7979	-0.43	$[-0.59, -0.21]$	0.0001	0.24	$[-0.05, 0.48]$	0.0402
PZ16a	0.78	$[0.69, 0.86]$	0.0000	-0.04	$[-0.30, 0.20]$	0.7157	0.72	$[0.60, 0.82]$	0.0000
PZ16b	0.85	$[0.77, 0.90]$	0.0000	0.18	$[-0.05, 0.37]$	0.0629	0.84	$[0.77, 0.89]$	0.0000
PZ17	0.35	$[0.17, 0.51]$	0.0002	0.43	$[0.25, 0.59]$	0.0000	0.62	$[0.48, 0.72]$	0.0000
PZ18	0.68	$[0.56, 0.77]$	0.0000	0.26	$[0.02, 0.47]$	0.0250	0.66	$[0.49, 0.77]$	0.0000
PZ19	0.32	$[0.06, 0.56]$	0.0069	0.19	$[-0.06, 0.43]$	0.1263	0.23	$[-0.04, 0.46]$	0.0616
PZ20	0.09	$[-0.17, 0.35]$	0.5000	-0.15	$[-0.38, 0.11]$	0.2359	-0.10	$[-0.32, 0.14]$	0.4326
PZ22	-0.44	$[-0.67, -0.15]$	0.0013	-0.43	$[-0.63, -0.14]$	0.0020	-0.37	$[-0.62, -0.05]$	0.0086
PZ23	0.28	$[-0.17, 0.63]$	0.1051	-0.11	$[-0.48, 0.30]$	0.5118	-0.13	$[-0.49, 0.28]$	0.4529
PZ30	-0.21	$[-0.51, 0.14]$	0.2432	-0.62	$[-0.78, -0.33]$	0.0001	-0.44	$[-0.67, -0.13]$	0.0093
PZ32	0.40	$[0.21, 0.56]$	0.0000	0.14	$[-0.04, 0.32]$	0.1395	0.61	$[0.47, 0.71]$	0.0000
PZ34	0.42	$[0.23, 0.59]$	0.0000	0.56	$[0.40, 0.72]$	0.0000	0.41	$[0.21, 0.58]$	0.0000
PZ36	0.92	$[0.83, 0.96]$	0.0000	0.42	$[0.10, 0.70]$	0.0080	0.86	$[0.74, 0.92]$	0.0000
PZ37	0.32	$[0.12, 0.50]$	0.0024	-0.27	$[-0.47, -0.04]$	0.0103	0.68	$[0.56, 0.79]$	0.0000
PZ38	-0.04	$[-0.24, 0.18]$	0.7497	0.10	$[-0.15, 0.33]$	0.3820	-0.03	$[-0.24, 0.16]$	0.7612

Table S4: Statistical validation of RNA3DCNN ranking performance across RNA-Puzzles-Bench targets under three training setups. The table presents the original Spearman rank correlation (r_s), 95% bootstrapped confidence intervals (CIs) from 1,000 resamples, and the associated p-values, comparing the RNA3DCNN architecture trained on lociPARSE, ARES, and RNA3DQA datasets.

**Puzzle**	**Trained on ARES**			**Trained on lociPARSE**			**Trained on RNA3DQA**		
	r_s	**95% CI**	p**-value**	r_s	**95% CI**	p**-value**	r_s	**95% CI**	p**-value**
PZ5	-0.21	$[-0.46, 0.05]$	0.1407	0.25	$[-0.11, 0.56]$	0.2242	-0.19	$[-0.53, 0.18]$	0.3757
PZ6	-0.24	$[-0.44, -0.02]$	0.0528	0.35	$[0.04, 0.59]$	0.0436	-0.22	$[-0.51, 0.10]$	0.2153
PZ7	-0.24	$[-0.41, -0.05]$	0.0153	0.26	$[-0.03, 0.51]$	0.0598	-0.17	$[-0.39, 0.10]$	0.2286
PZ9	-0.17	$[-0.38, 0.07]$	0.1566	-0.49	$[-0.72, -0.12]$	0.0030	-0.34	$[-0.62, 0.01]$	0.0524
PZ14Bound	-0.28	$[-0.45, -0.11]$	0.0015	0.31	$[0.06, 0.53]$	0.0142	0.04	$[-0.22, 0.32]$	0.7703
PZ14Free	-0.29	$[-0.46, -0.11]$	0.0028	0.38	$[0.11, 0.58]$	0.0053	0.06	$[-0.24, 0.34]$	0.6968
PZ15	0.20	$[0.07, 0.33]$	0.0032	-0.32	$[-0.49, -0.13]$	0.0009	0.07	$[-0.12, 0.25]$	0.5054
PZ15noend	0.12	$[-0.06, 0.30]$	0.1636	-0.25	$[-0.45, -0.01]$	0.0327	0.08	$[-0.13, 0.27]$	0.4867
PZ15rec	0.15	$[-0.01, 0.32]$	0.0659	-0.22	$[-0.45, 0.04]$	0.0555	-0.03	$[-0.26, 0.19]$	0.7961
PZ16a	-0.01	$[-0.15, 0.14]$	0.8918	0.06	$[-0.14, 0.25]$	0.5899	0.36	$[0.17, 0.51]$	0.0004
PZ16b	0.02	$[-0.12, 0.17]$	0.7723	0.25	$[0.04, 0.43]$	0.0114	0.26	$[0.05, 0.45]$	0.0083
PZ17	-0.08	$[-0.23, 0.06]$	0.2520	0.18	$[-0.02, 0.39]$	0.0602	-0.02	$[-0.23, 0.17]$	0.8475
PZ18	0.25	$[0.11, 0.39]$	0.0018	-0.16	$[-0.36, 0.07]$	0.1642	0.24	$[0.04, 0.42]$	0.0392
PZ19	-0.25	$[-0.40, -0.08]$	0.0035	0.50	$[0.29, 0.68]$	0.0000	-0.15	$[-0.40, 0.10]$	0.2089
PZ20	-0.14	$[-0.30, 0.03]$	0.1153	0.25	$[0.00, 0.50]$	0.0475	-0.19	$[-0.45, 0.08]$	0.1234
PZ22	-0.10	$[-0.30, 0.09]$	0.3182	-0.14	$[-0.41, 0.14]$	0.3332	-0.05	$[-0.35, 0.24]$	0.7103
PZ23	-0.15	$[-0.37, 0.10]$	0.2103	0.27	$[-0.14, 0.59]$	0.1127	0.03	$[-0.32, 0.39]$	0.8800
PZ30	-0.10	$[-0.35, 0.14]$	0.4132	-0.06	$[-0.44, 0.33]$	0.7596	0.07	$[-0.29, 0.44]$	0.6951
PZ32	-0.20	$[-0.32, -0.06]$	0.0043	0.19	$[0.01, 0.36]$	0.0492	-0.11	$[-0.30, 0.09]$	0.2733
PZ34	-0.35	$[-0.46, -0.21]$	0.0000	0.60	$[0.45, 0.72]$	0.0000	-0.22	$[-0.40, -0.01]$	0.0294
PZ36	-0.34	$[-0.55, -0.10]$	0.0027	0.18	$[-0.13, 0.51]$	0.2674	-0.23	$[-0.58, 0.14]$	0.1685
PZ37	0.16	$[0.00, 0.31]$	0.0307	-0.16	$[-0.37, 0.06]$	0.1392	0.12	$[-0.11, 0.35]$	0.2699
PZ38	0.02	$[-0.14, 0.18]$	0.8095	-0.08	$[-0.31, 0.17]$	0.4932	-0.03	$[-0.26, 0.19]$	0.7689

Table S5: Wilcoxon signed-rank test results comparing rank error of the original lociPARSE model versus RNA3DQA-trained lociPARSE against the reference ranking, for each of the 23 contests. Two one-sided alternatives are tested: lociPARSE error is less than RNA3DQA error, and RNA3DQA error is less than lociPARSE error.

		H_1: lociPARSE < RNA3DQA		H_1: RNA3DQA < lociPARSE	
Puzzle	W	p-value	Sig.	p-value	Sig.
PZ5	81.00	0.0242	✓	0.9758	
PZ6	168.00	0.0921		0.9079	
PZ7	523.50	0.1879		0.8121	
PZ9	143.50	0.0197	✓	0.9803	
PZ14free	434.00	0.0843		0.9157	
PZ14bound	453.50	0.0003	✓	0.9997	
PZ15	4262.50	1.0000		0.0000	✓
PZ15rec	1837.00	0.9923		0.0077	✓
PZ15noend	1853.50	0.9940		0.0060	✓
PZ16a	2016.50	0.3810		0.6190	
PZ16b	2316.50	0.1503		0.8497	
PZ17	4007.50	0.9989		0.0011	✓
PZ18	1409.50	0.5472		0.4528	
PZ19	826.00	0.0170	✓	0.9830	
PZ20	894.00	0.1217		0.8783	
PZ22	694.00	0.7073		0.2927	
PZ23	213.00	0.0473	✓	0.9527	
PZ30	210.50	0.0683		0.9317	
PZ32	3523.50	0.9911		0.0089	✓
PZ34	2628.50	0.6390		0.3610	
PZ36	212.00	0.0284	✓	0.9716	
PZ37	3033.50	1.0000		0.0000	✓
PZ38	1773.50	0.4795		0.5205	

✓ indicates $p < 0.05$ (statistically significant, reject H_0).

Figure S1: Predictive performance shifts in ARES, lociPARSE, and RNA3DCNN models evaluated on 23 RNA-Puzzles benchmark units. Vertical lines denote the baseline Spearman correlation with ground truth for the original (off-the-shelf) models. Colored dots represent the performance of the respective models after retraining on the RNA3DQA training set.

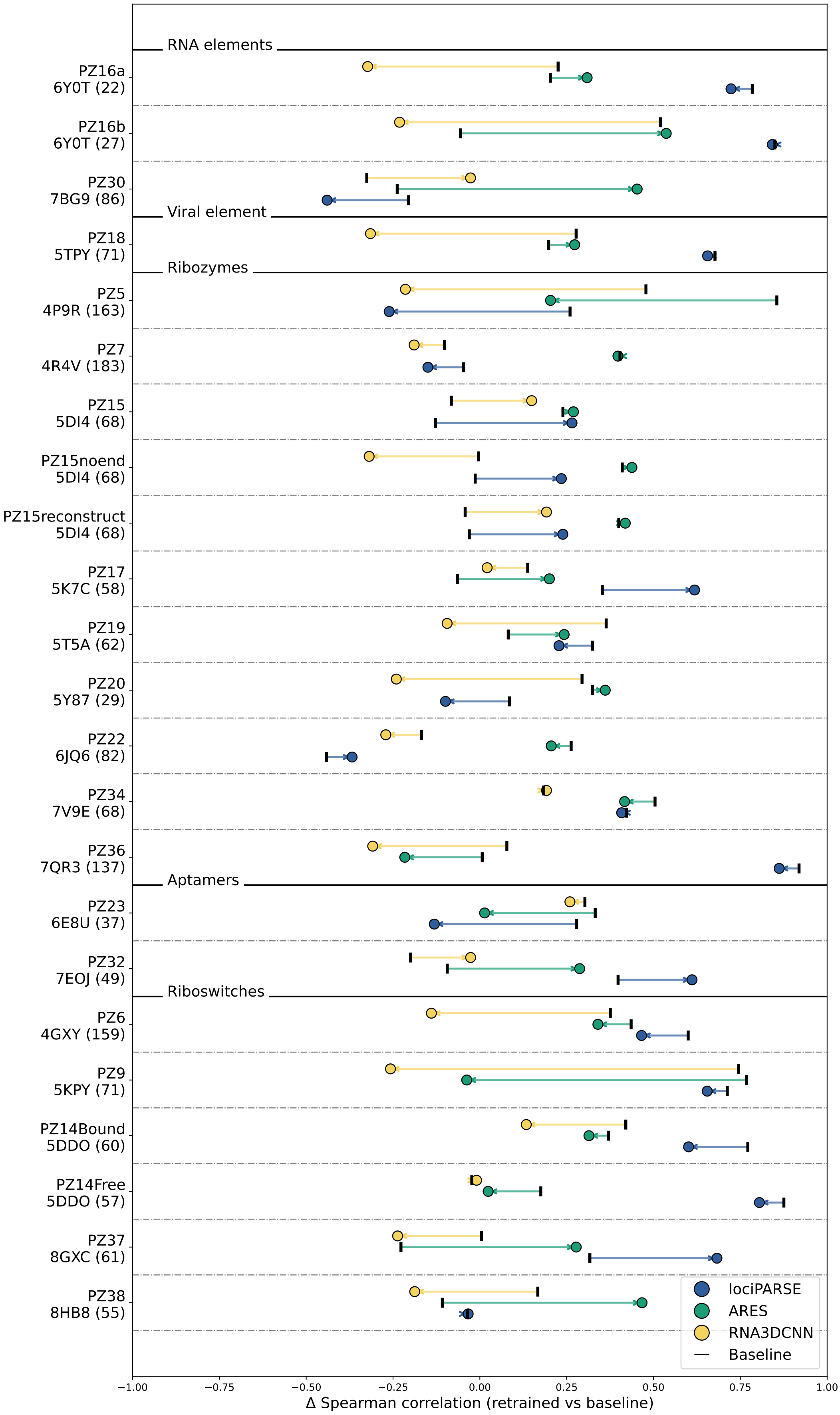

Figure S2: Effect of training dataset on the predictive performance of ARES across 23 RNA-Puzzles benchmark units. Vertical lines denote the baseline Spearman correlation of the original (off-the-shelf) model. Colored dots represent ARES retrained on the lociPARSE and RNA3DQA training datasets, whereas a star denotes ARES retrained on its native training dataset.

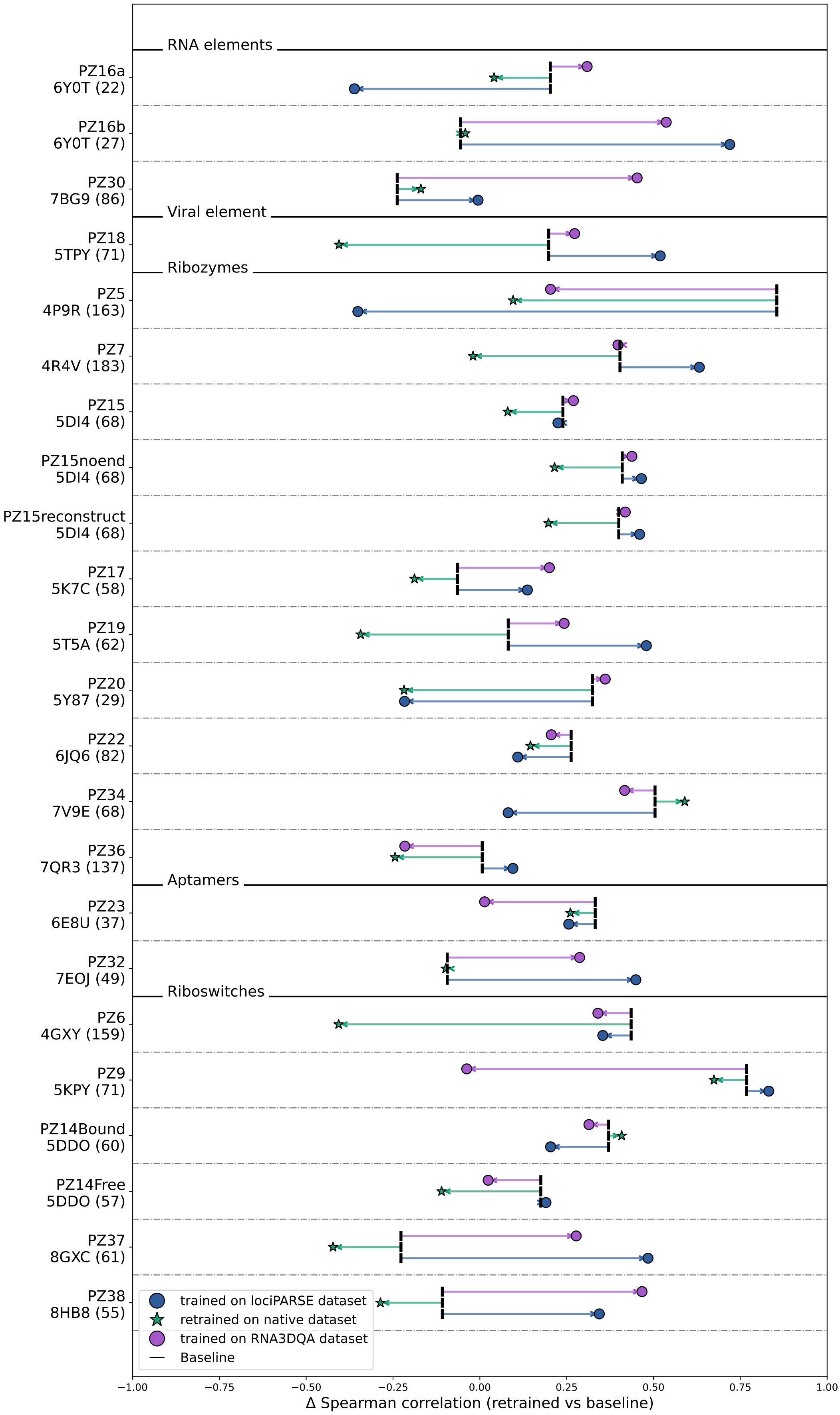

Figure S3: Effect of training dataset on the predictive performance of lociPARSE across 23 RNA-Puzzles benchmark units. Vertical lines denote the baseline Spearman correlation of the original (off-the-shelf) model. Colored dots represent lociPARSE retrained on the ARES and RNA3DQA training datasets, whereas a star denotes lociPARSE retrained on its native training dataset.

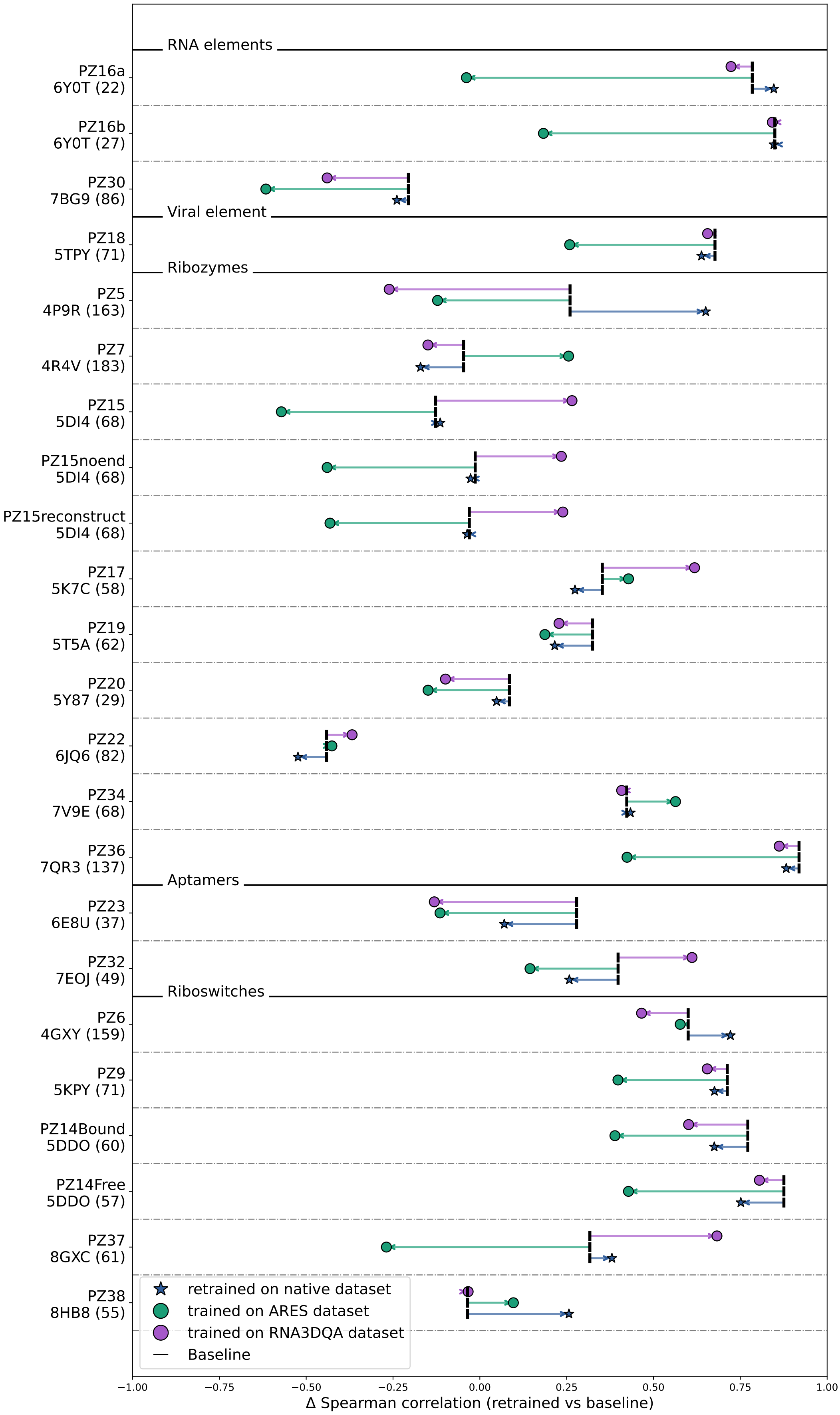

Figure S4: Effect of training dataset on the predictive performance of RNA3DCNN across 23 RNA-Puzzles benchmark units. Vertical lines denote the baseline Spearman correlation of the original (off-the-shelf) model. Colored dots represent RNA3DCNN retrained on the ARES, lociPARSE, and RNA3DQA training datasets; a star denotes off-the-shelf RNA3DCNN_MDMC model.

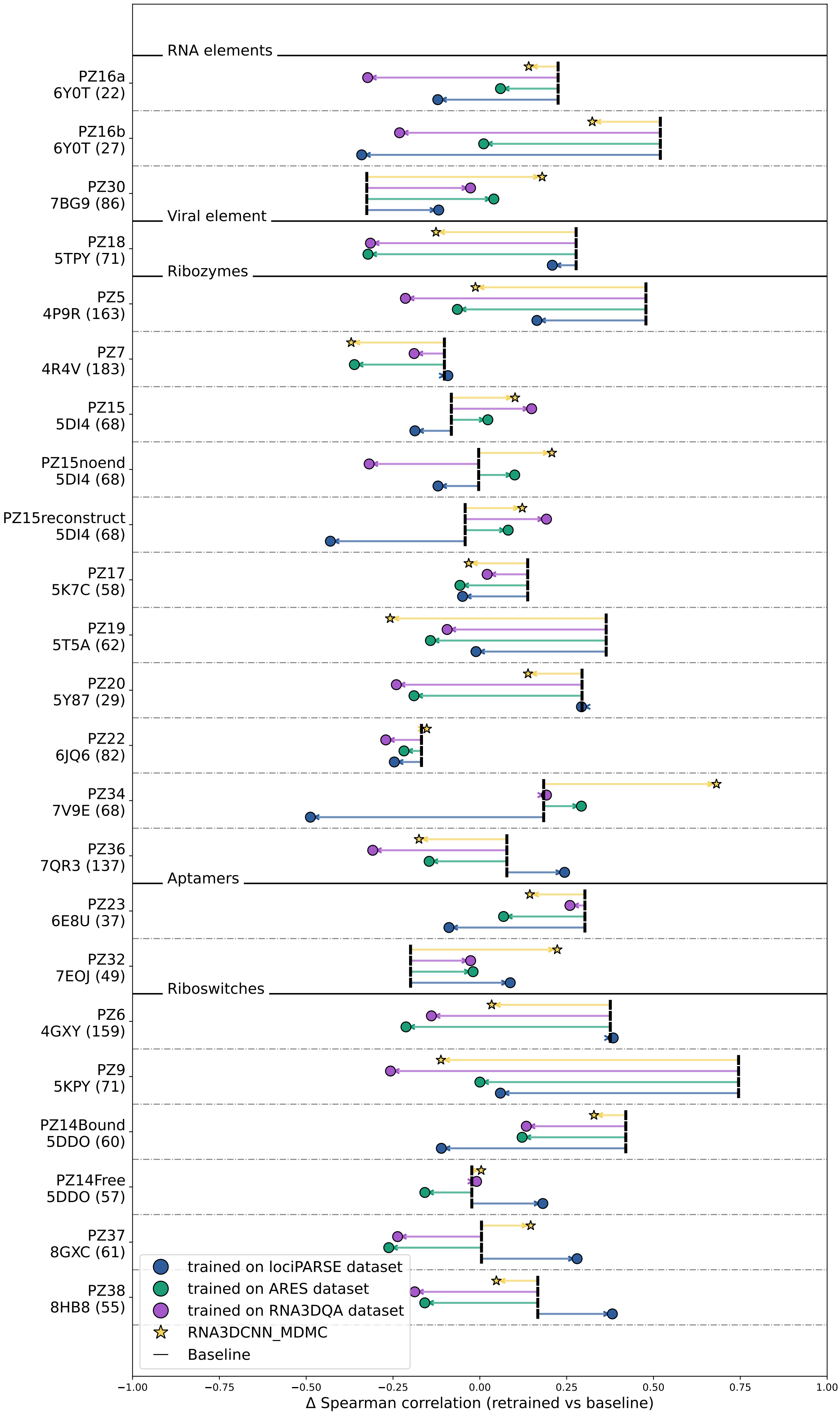

Figure S5: Prediction accuracy of RNA 3D structure quality assessment models. Each histogram shows the distribution of Spearman correlation coefficients on RNA-Puzzles-Bench across combinations of architecture (columns) and training set (rows).

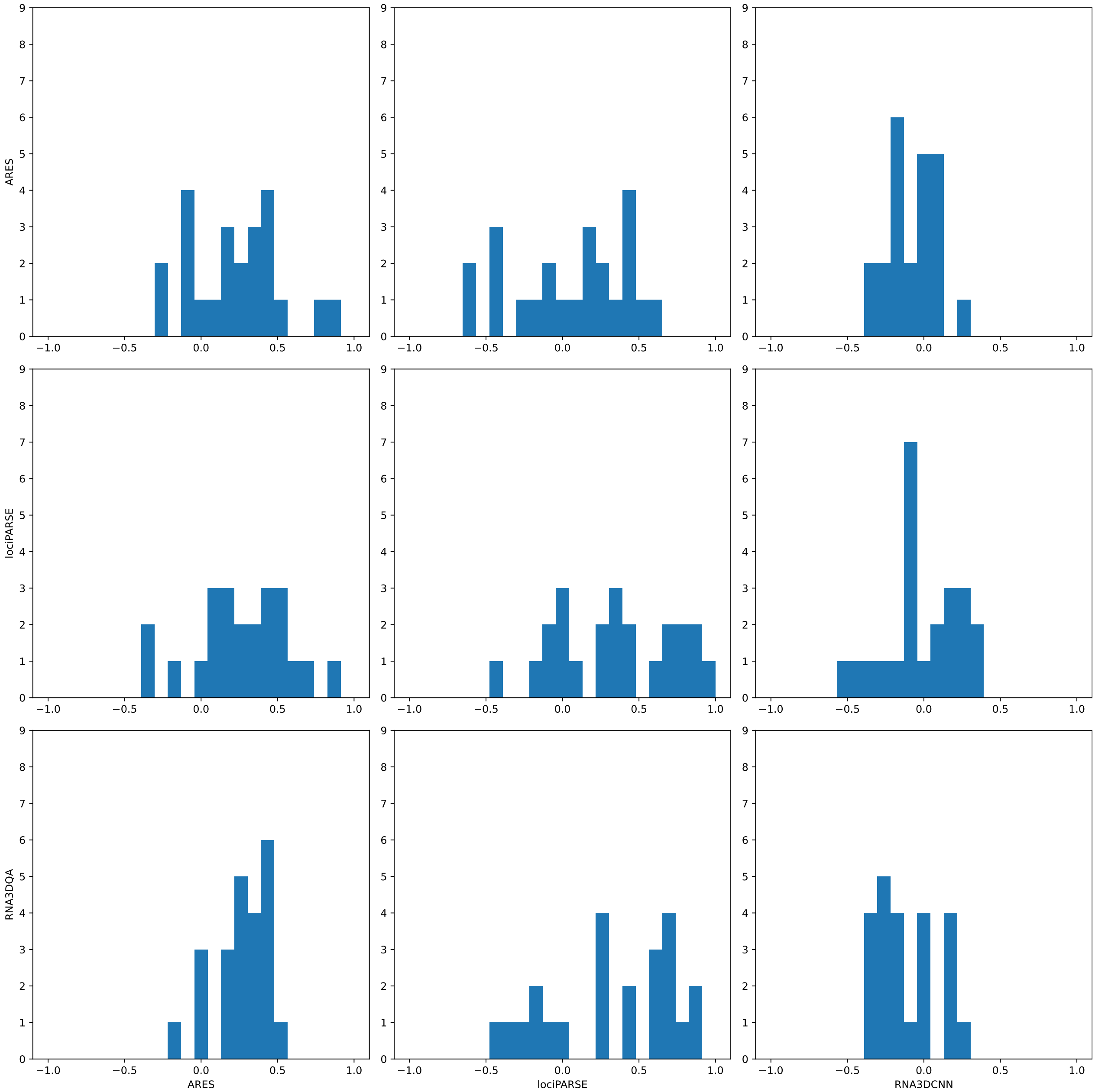

Figure S6: Prediction accuracy of RNA structure quality assessment models. Each histogram shows the distribution of Spearman correlation coefficients on RNA-Puzzles-Bench, based on predictions from off-the-shelf models.

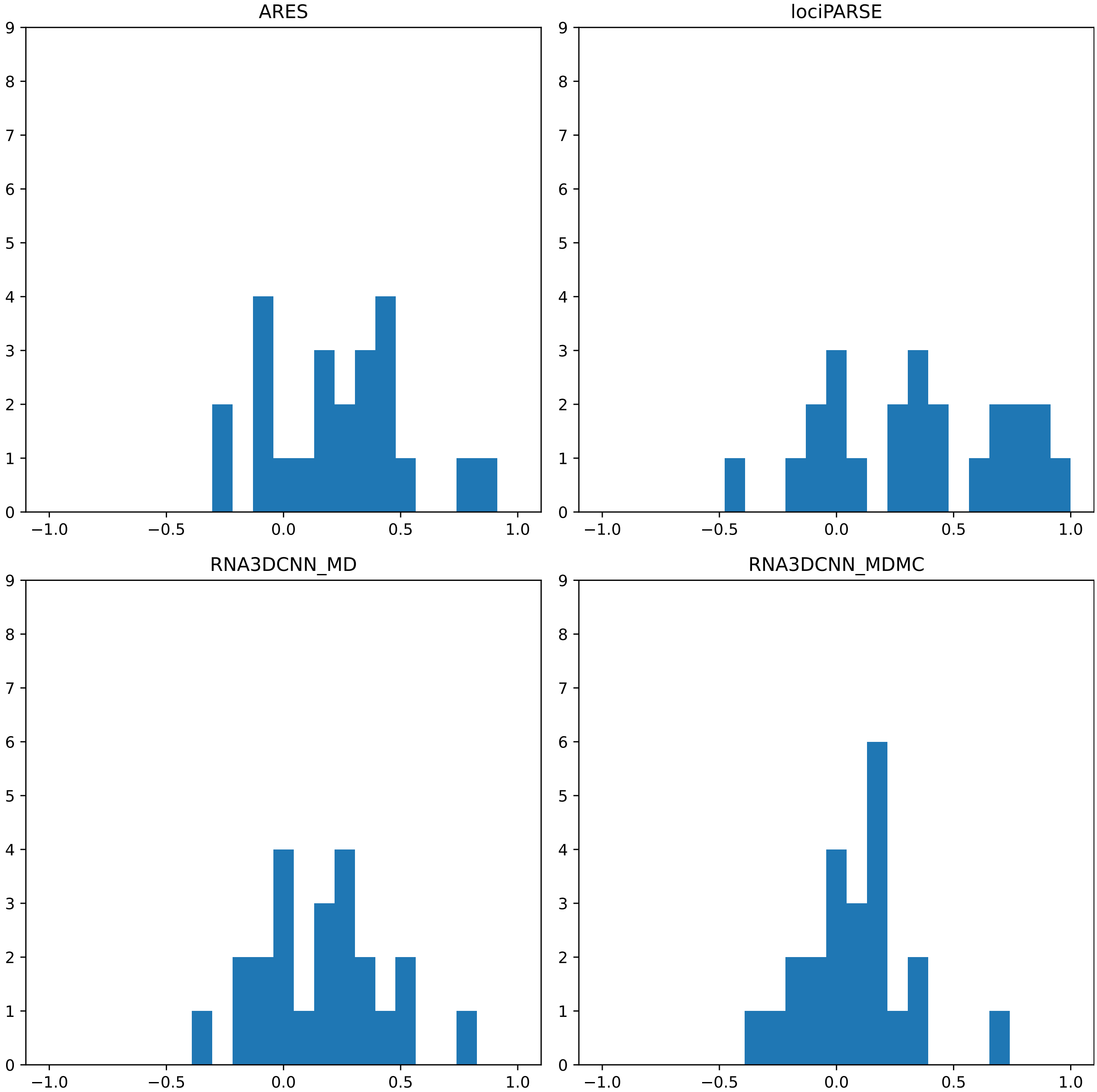

Figure S7: Distribution of experimental RNA 3D structures in the lociPARSE training set by Rfam family.

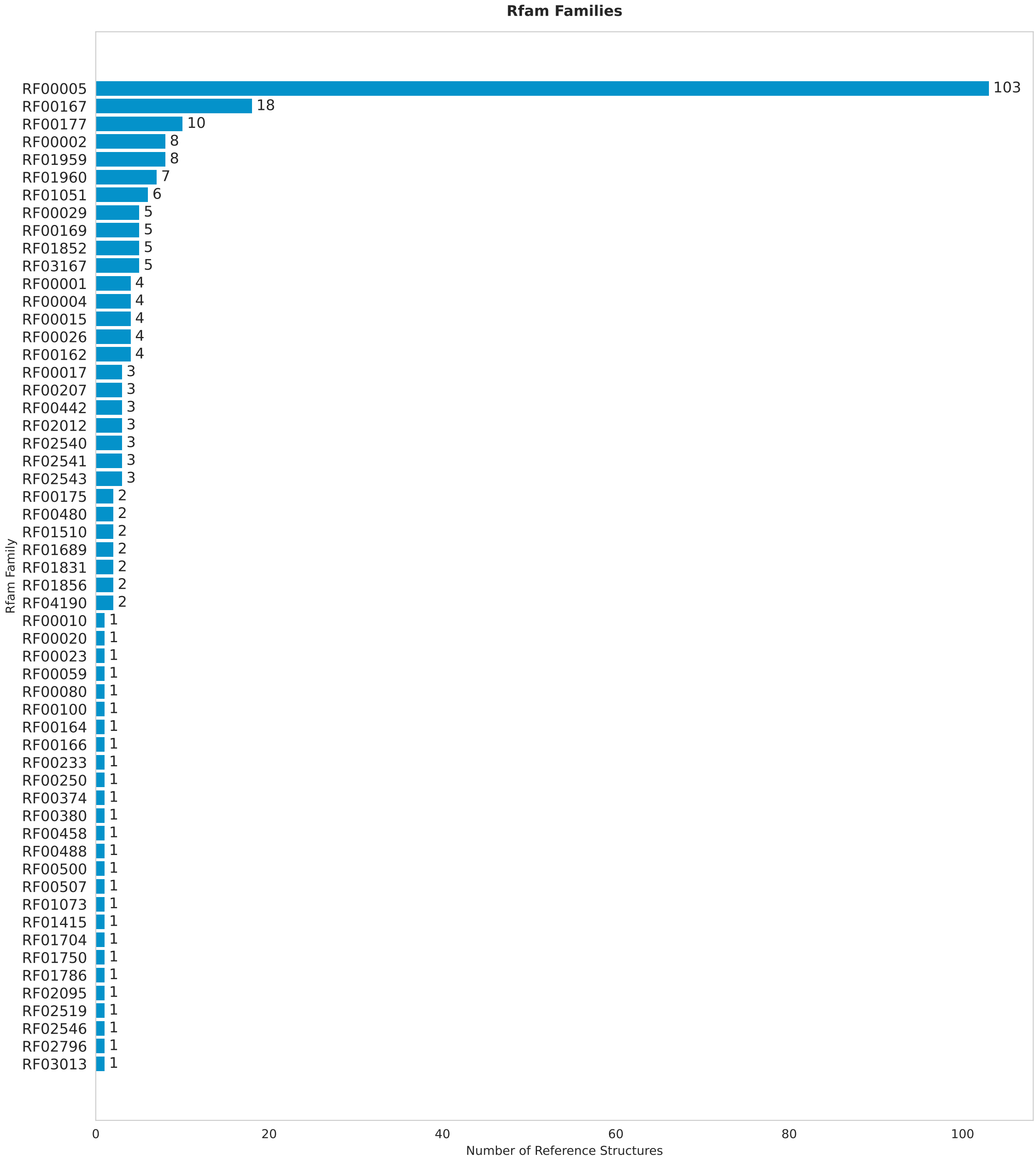

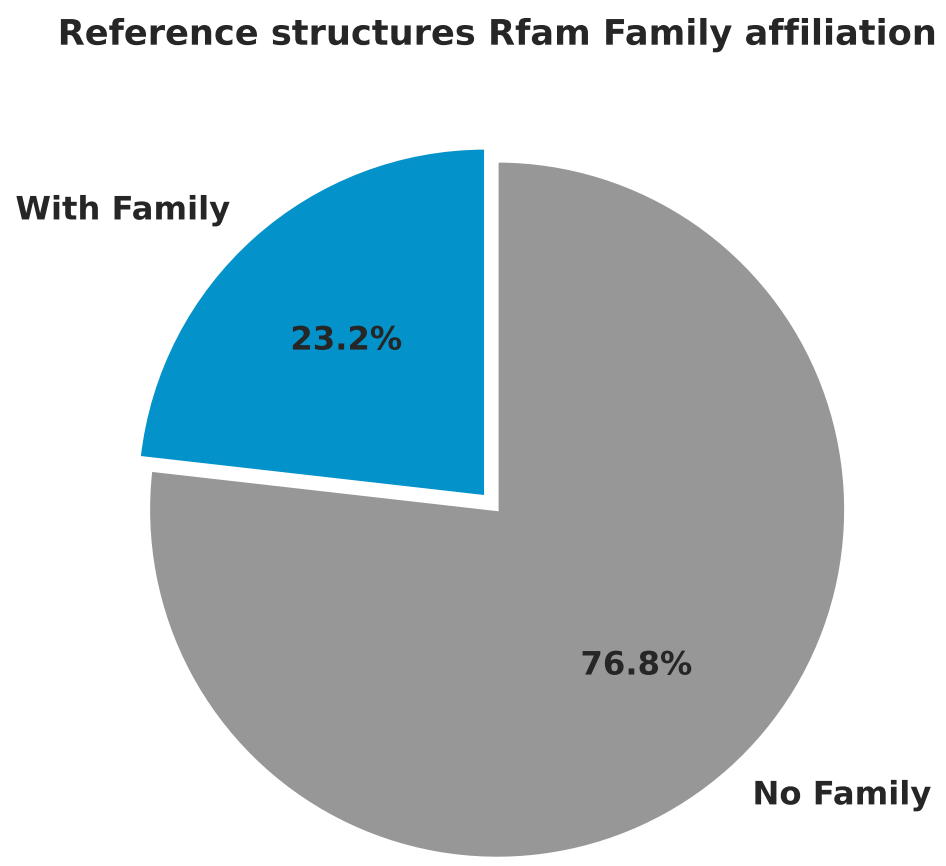

Figure S8: Distribution of experimental RNA 3D structures in the lociPARSE validation set by Rfam family.

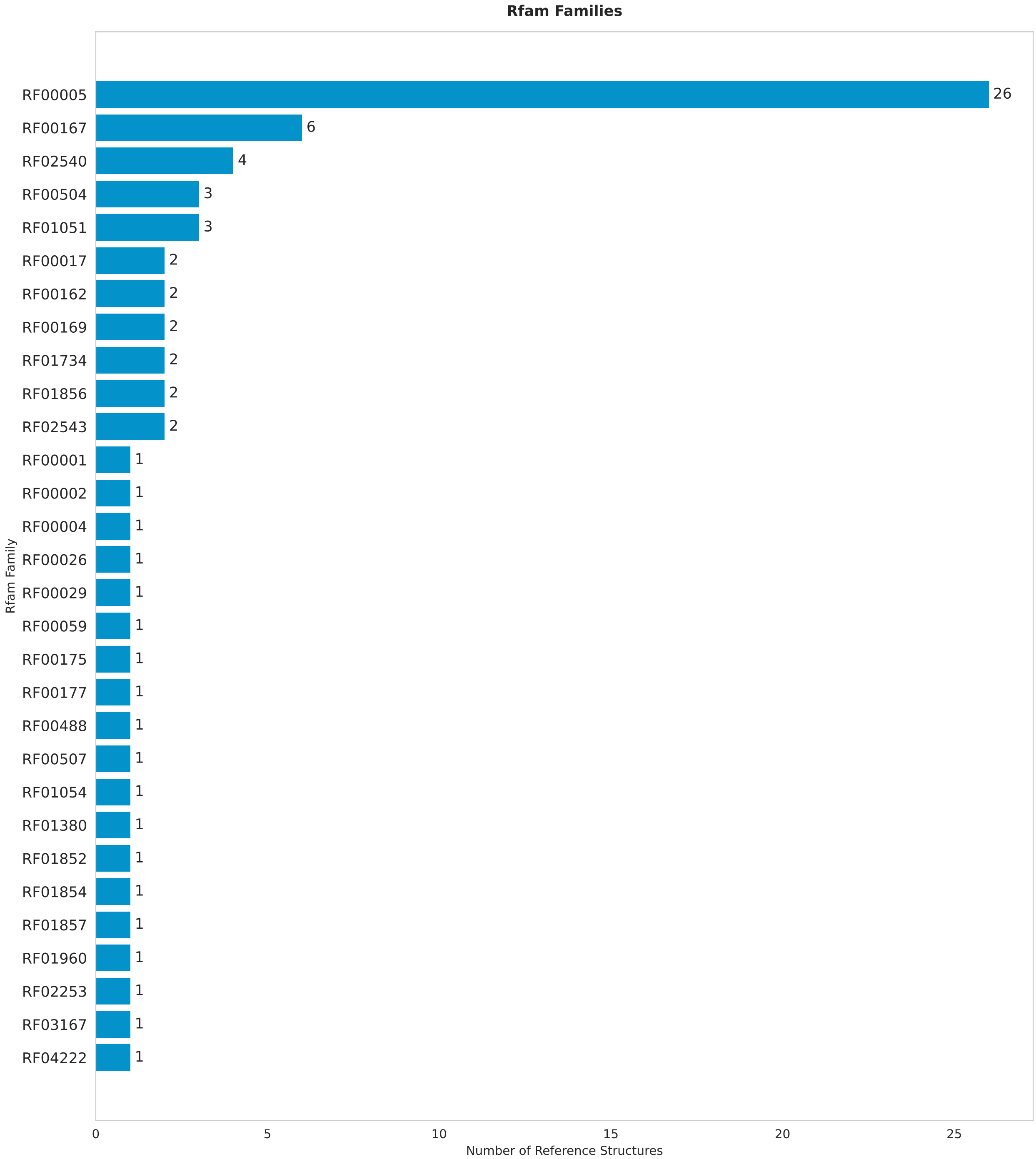

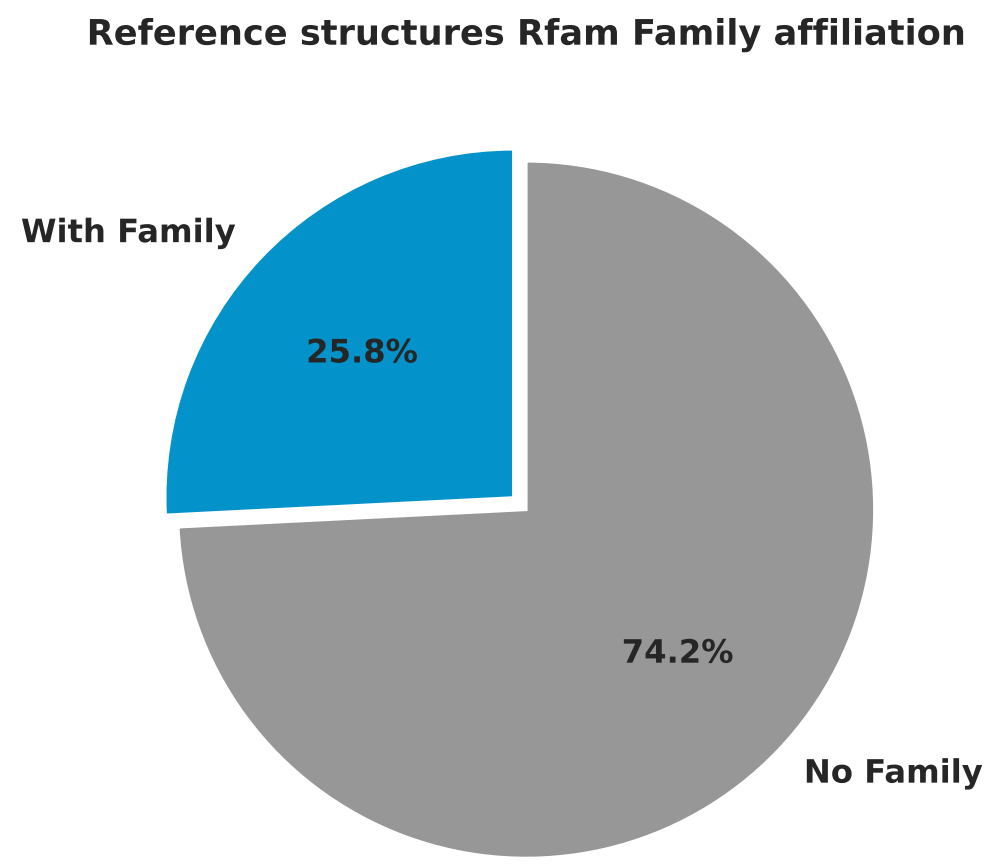

Figure S9: Quantitative characterization of two training datasets restricted to high-quality structural models. Column 1 (Panels A and C) shows the lociPARSE dataset, and Column 2 (Panels B and D) represents the RNA3DQA dataset, with the analysis restricted exclusively to decoy structures with reference RMSD values below 16 Å. Panels A and B show the distribution of decoy models across different sequence lengths (reflecting the target sizes in the underlying prediction pools), while Panels C and D plot decoy RMSD against RNA sequence length to show the diverse spread of model qualities across different length scales.

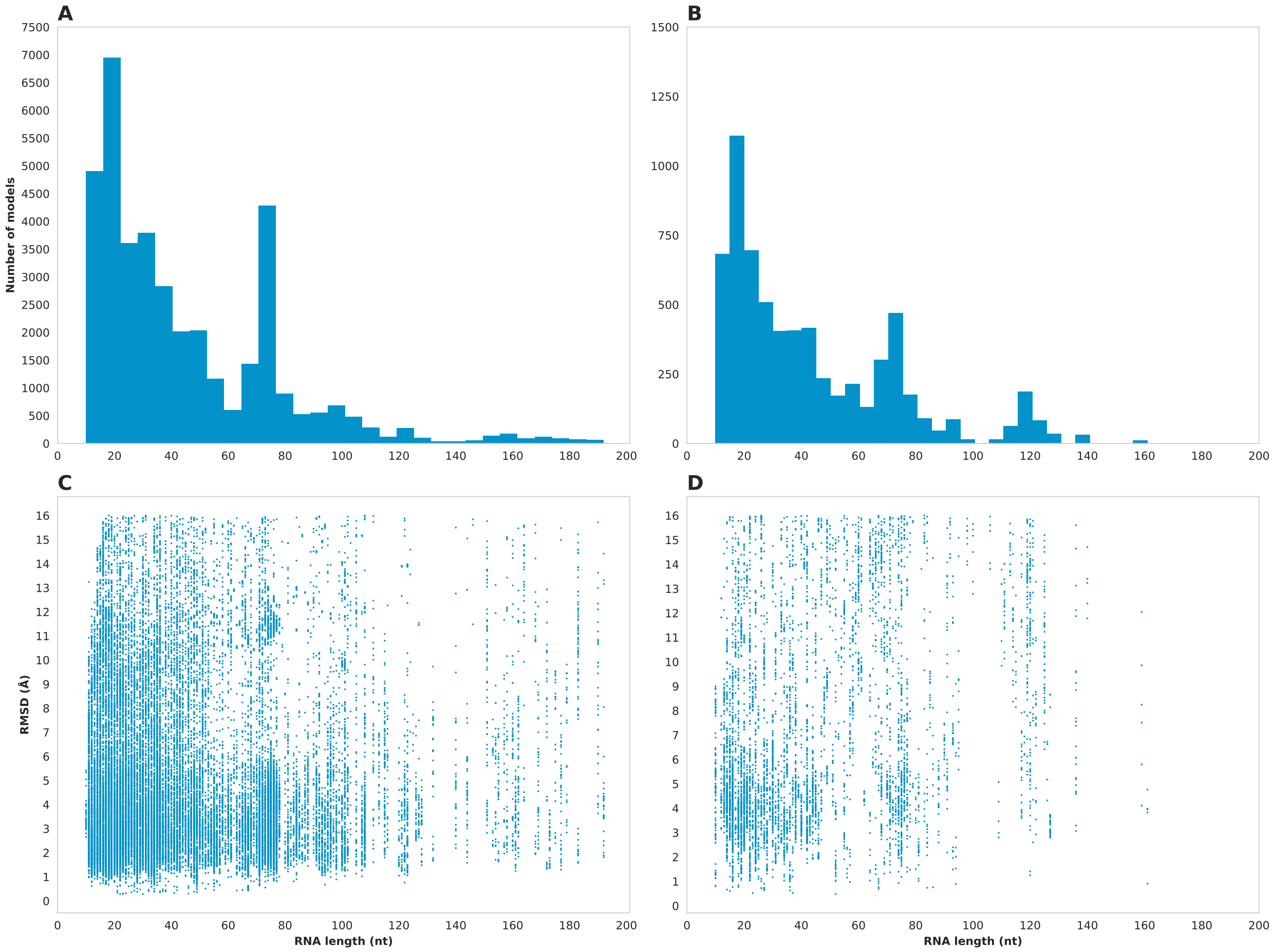

Figure S10: Distribution of experimental RNA 3D structures in the RNA3DQA training set by Rfam family.

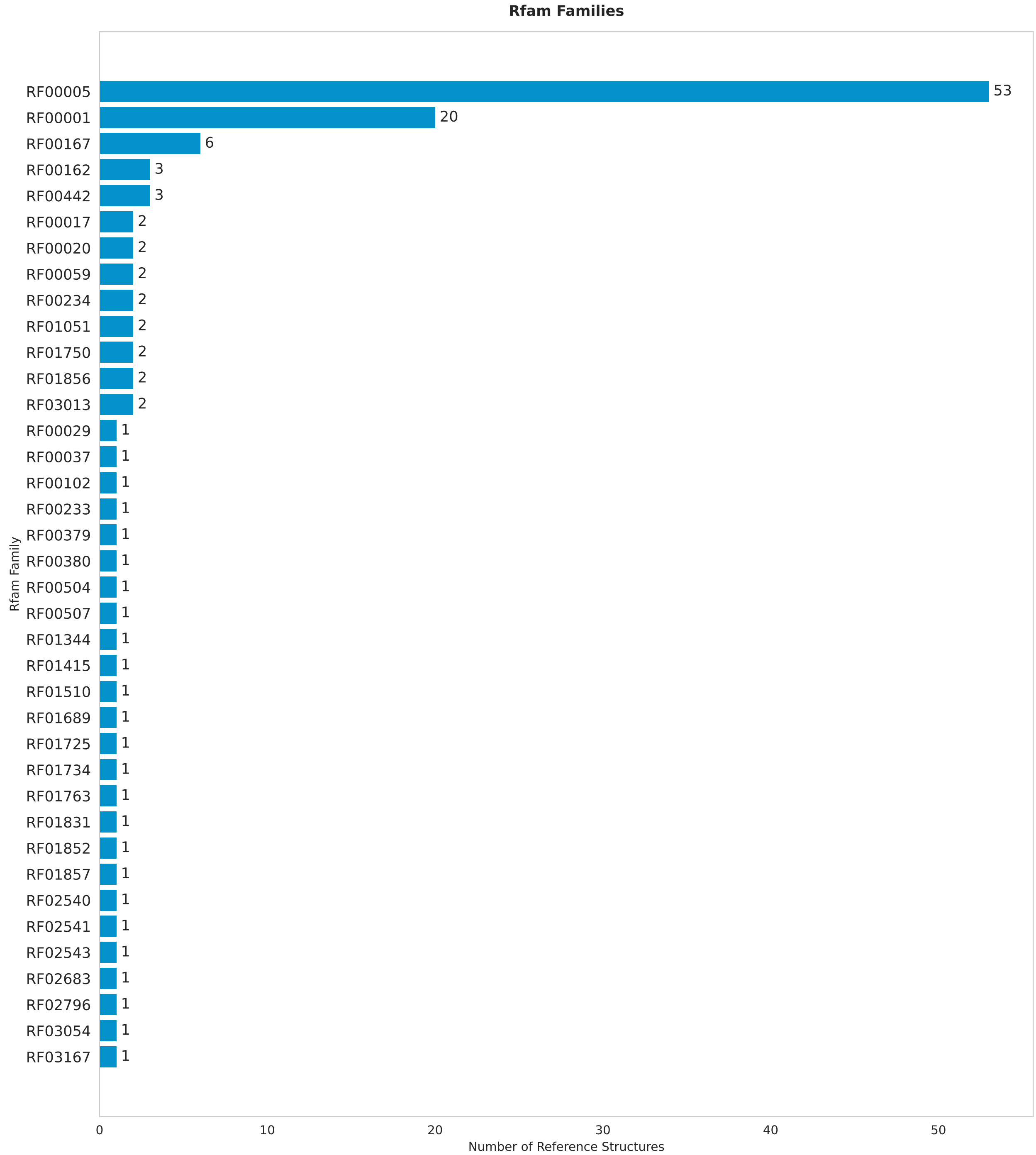

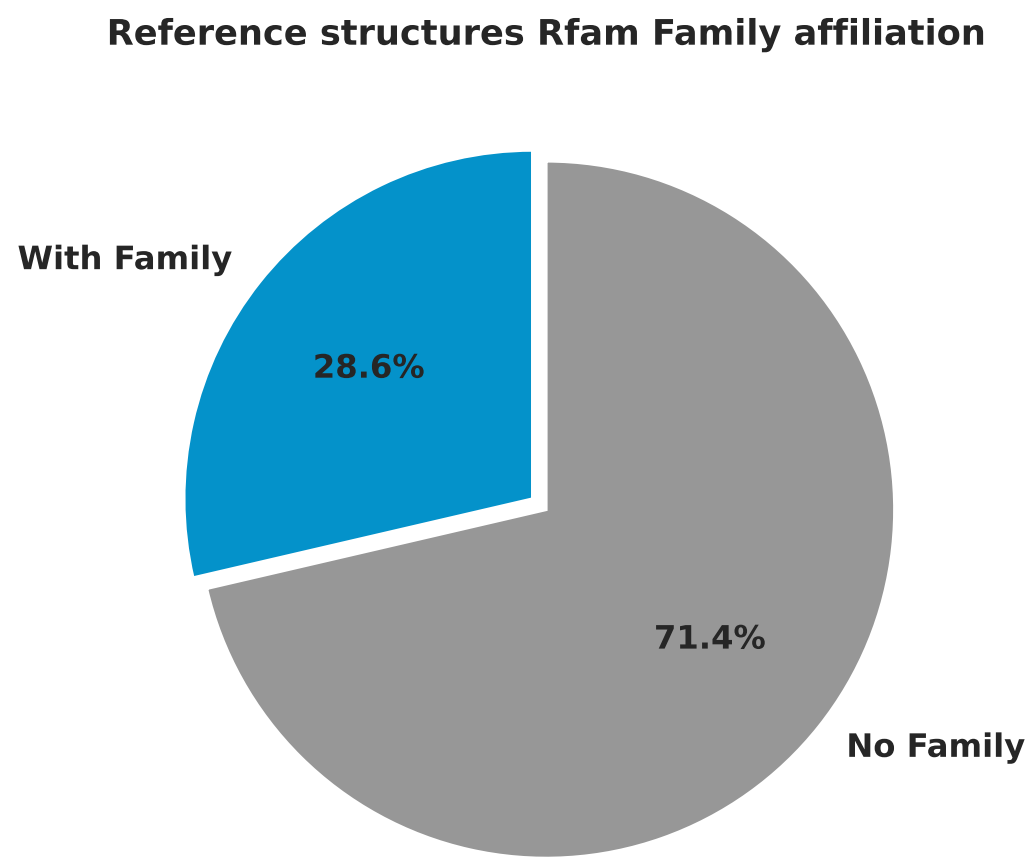

Figure S11: Distribution of experimental RNA 3D structures in the RNA3DQA validation set by Rfam family.

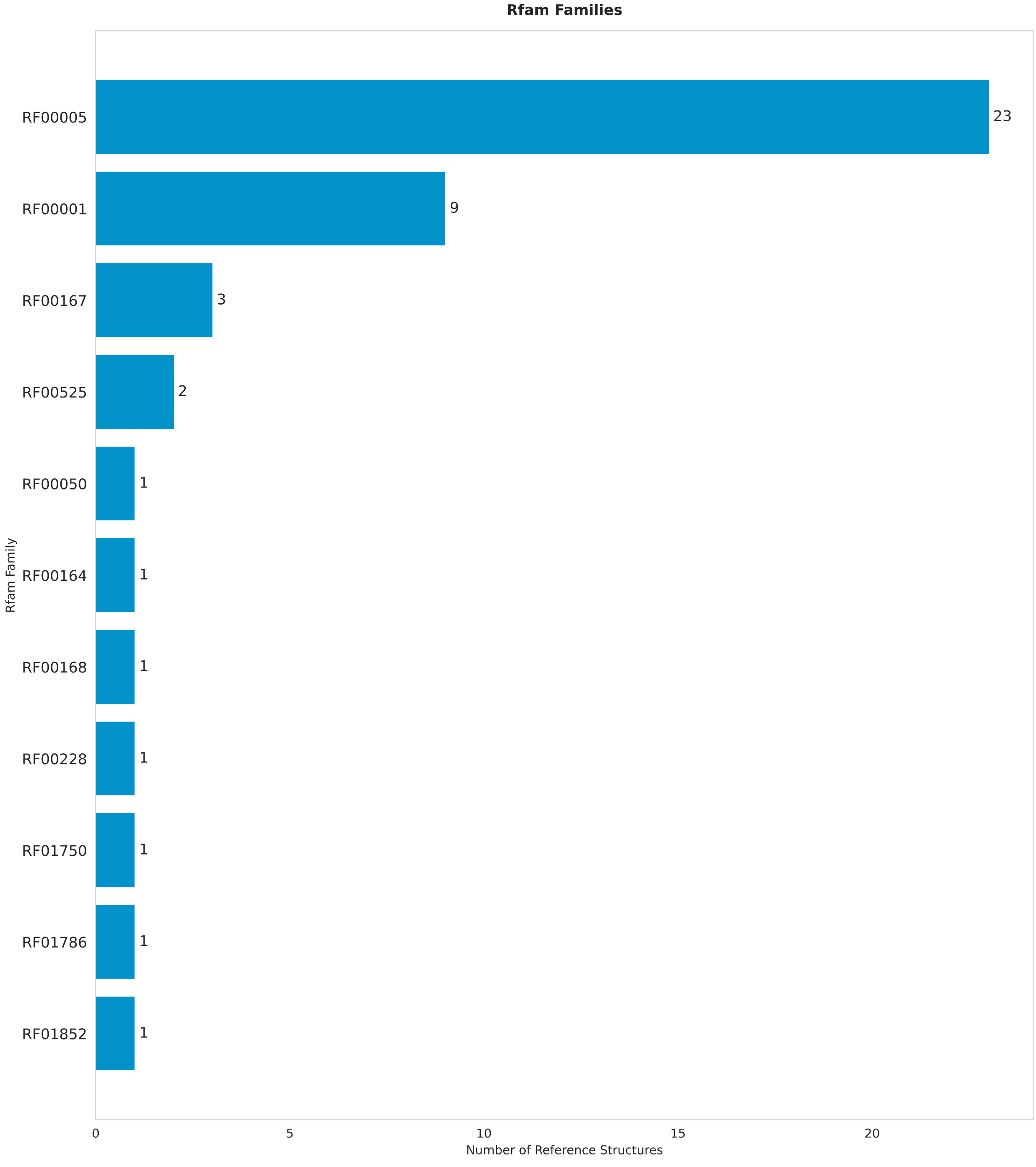

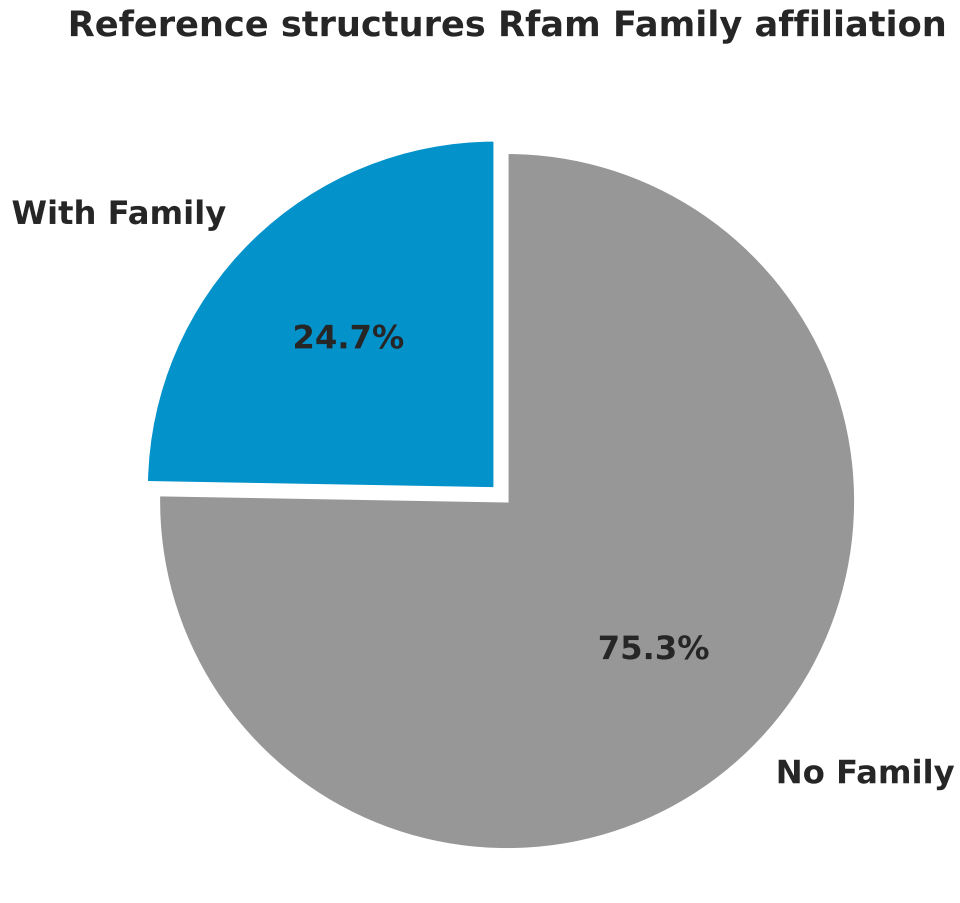

Figure S12: Global distribution of absolute rank errors for the ARES architecture across four training setups (original off-the-shelf, native-retrained, lociPARSE-trained, and RNA3DQA-trained), evaluated over the full RNA-Puzzles-Bench dataset (1,546 predicted models) partitioned into three model quality groups (top, middle, and bottom third) based on original RMSD values.

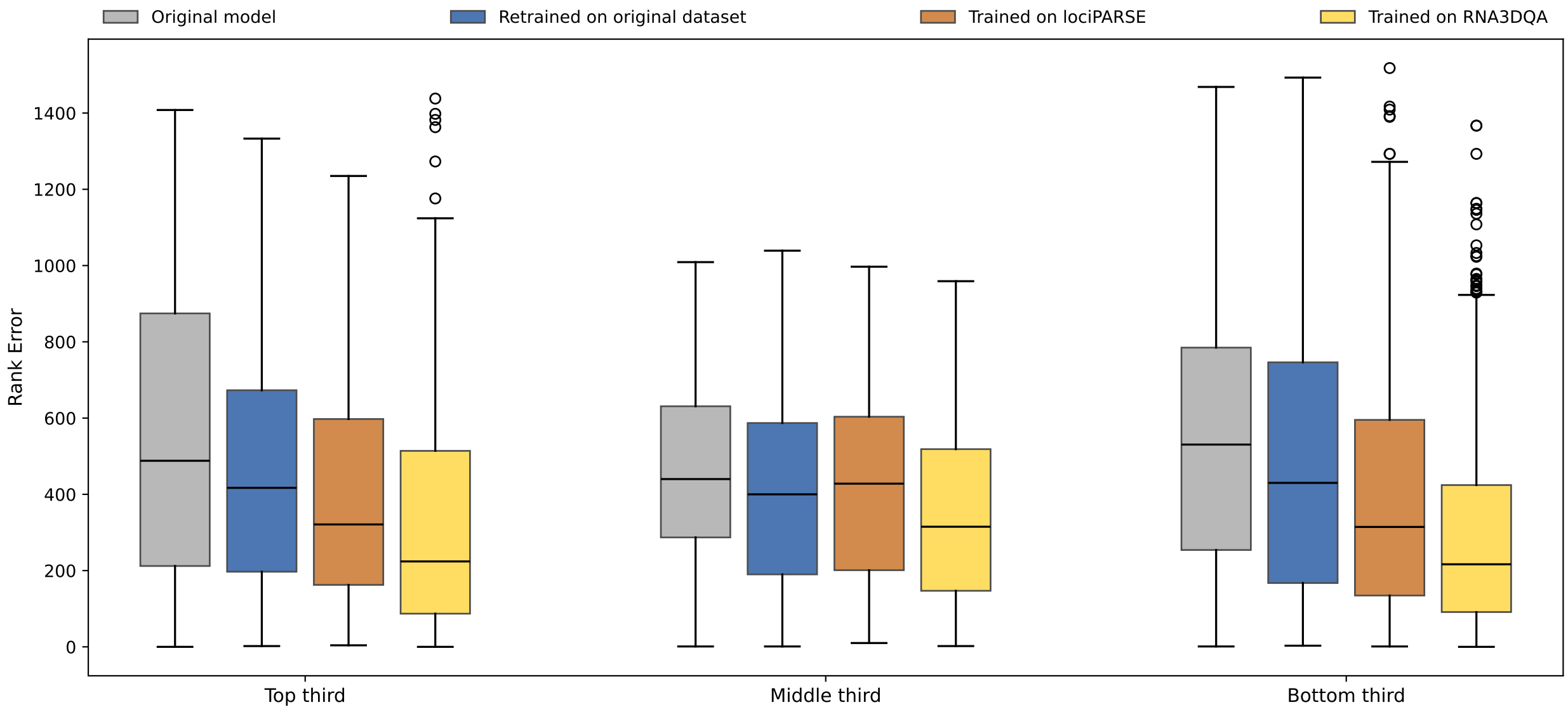

Figure S13: Global distribution of absolute rank errors for the lociPARSE architecture across four training setups (original off-the-shelf, native-retrained, ARES-trained, and RNA3DQA-trained), evaluated over the full RNA-Puzzles-Bench dataset (1,546 predicted models) partitioned into three model quality groups (top third, middle third, bottom third) based on original lDDT values.

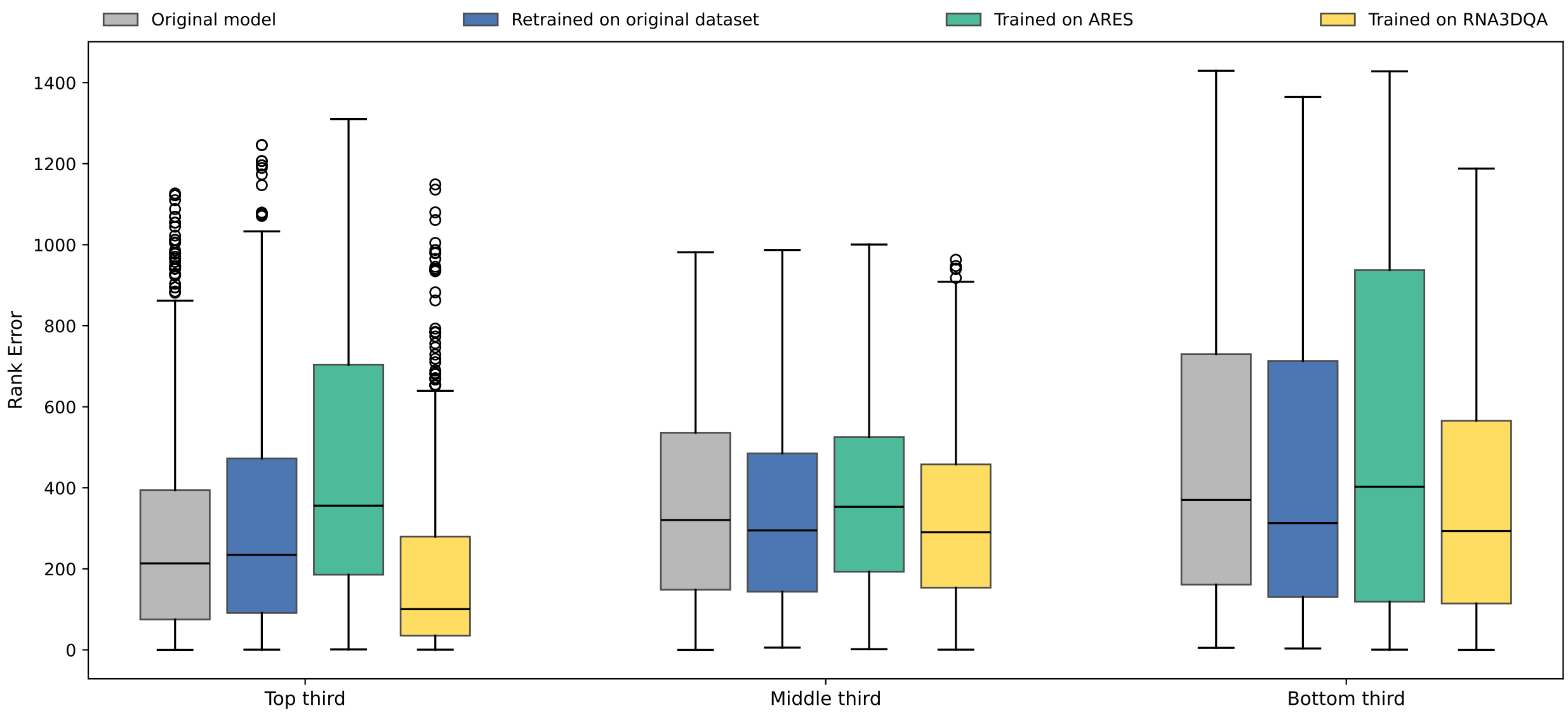

Figure S14: Global distribution of absolute rank errors for the RNA3DCNN architecture across five training setups (original off-the-shelf, RNA3DCNN_MDMC-trained, ARES-trained, lociPARSE-trained, and RNA3DQA-trained), evaluated over the full RNA-Puzzles-Bench dataset (1,546 predicted models) partitioned into three model quality groups (top third, middle third, bottom third) based on original RMSD values.

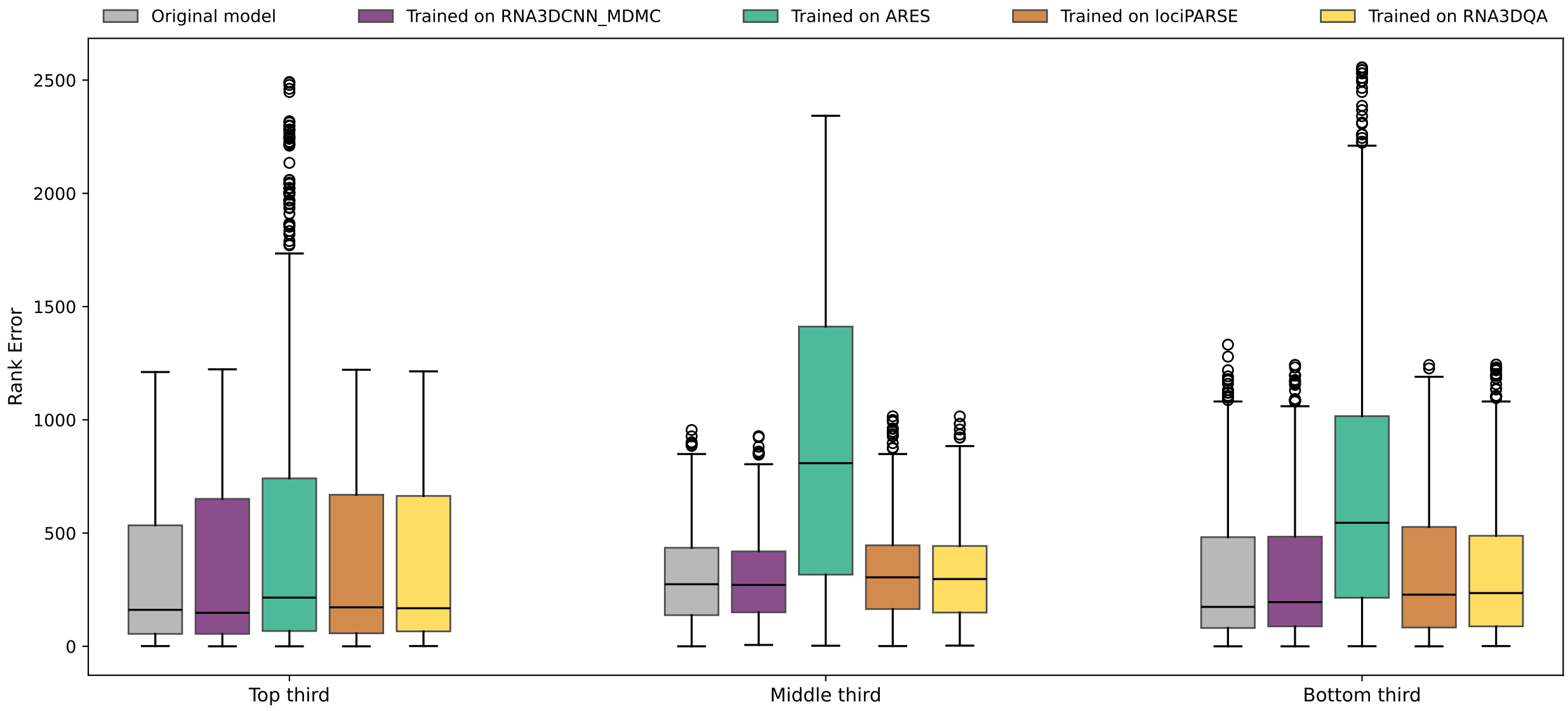

Figure S15: Performance variance (Spearman rank correlations) of the lociPARSE architecture retrained on the RNA3DQA dataset across four independent random seeds, evaluated on the complete RNA-Puzzles-Bench dataset (23 benchmark units, 1,546 predicted 3D structural models). Boxplots display the median (center line) and interquartile range (IQR, box boundaries).

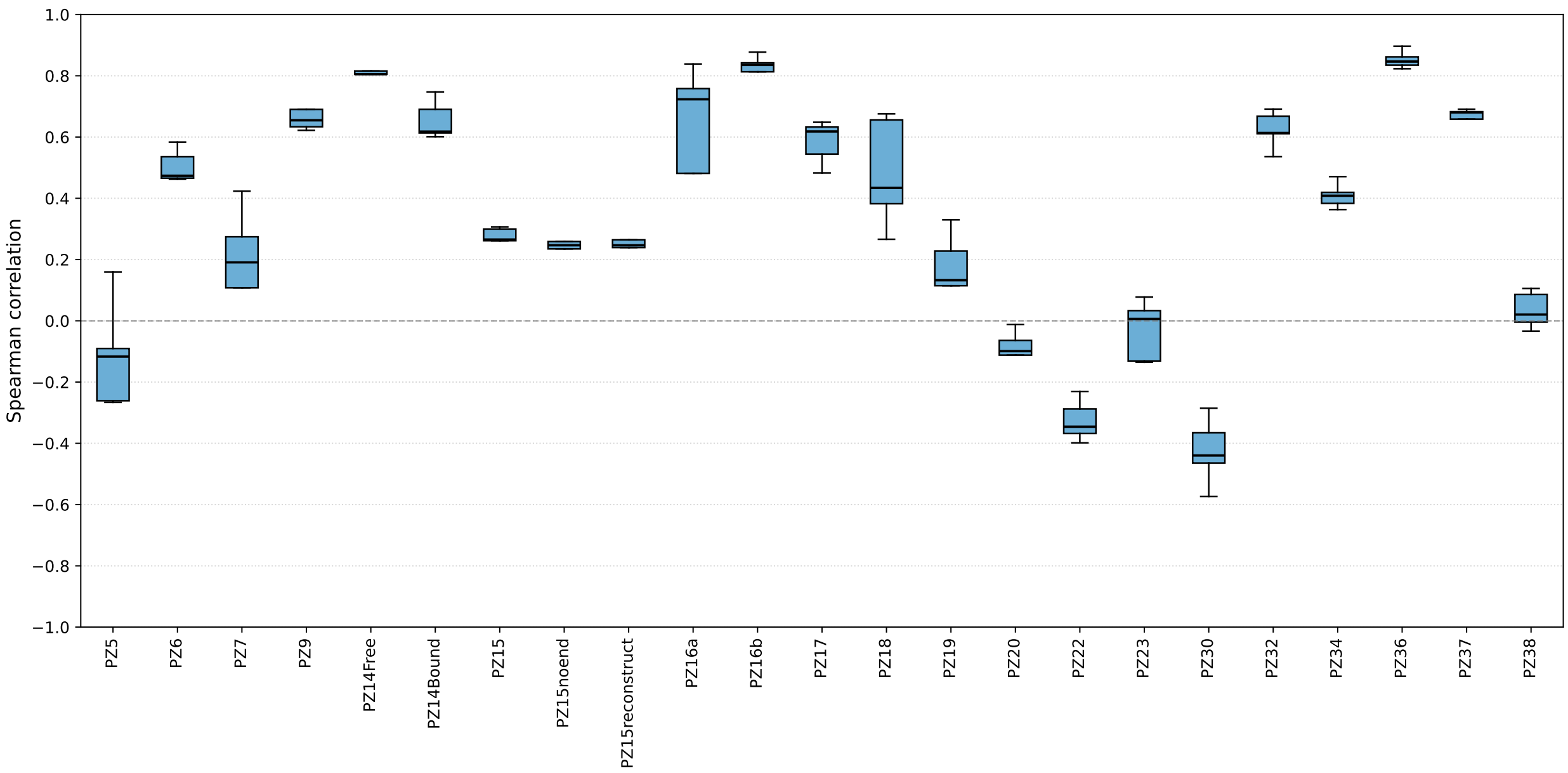

Figure S16: Performance variance (Spearman rank correlations) of the RNA3DCNN architecture retrained on the RNA3DQA dataset across four independent random seeds, evaluated on the complete RNA-Puzzles-Bench dataset (23 benchmark units, 1,546 predicted 3D structural models). Boxplots display the median (center line) and interquartile range (IQR, box boundaries).

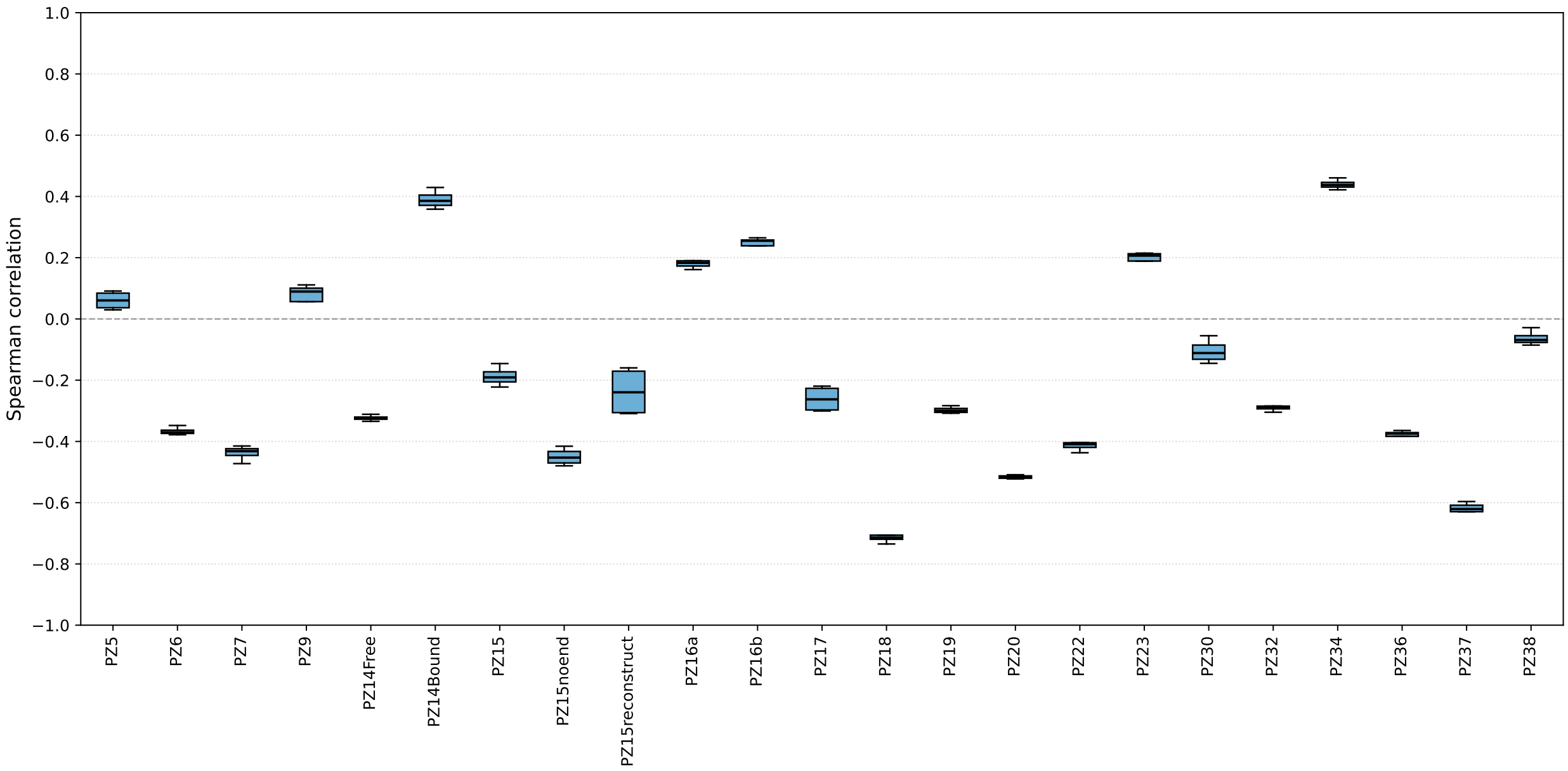

Supplement: Supplementary file 1 [file adamczyk_supplementary_bbag630.pdf]
